# Establishment of human periodontal ligament cell lines with *ALPL* mutations to mimic dental aspects of hypophosphatasia

**DOI:** 10.3389/fcell.2025.1572571

**Published:** 2025-06-03

**Authors:** Jana Schiffmaier, Sofia Rehling, Katharina Marnet, Angela Borst, Drenka Trivanović, Denitsa Docheva, Franz Jakob, Stephanie Graser, Marietta Herrmann, Daniel Liedtke

**Affiliations:** ^1^ IZKF Group Tissue Regeneration in Musculoskeletal Diseases, University Hospital Würzburg, Würzburg, Germany; ^2^ Bernhard-Heine-Center for Locomotion Research, University of Würzburg, Würzburg, Germany; ^3^ Institute of Human Genetics, Biocenter, University of Würzburg, Würzburg, Germany; ^4^ Institute for Medical Research, National Institute of the Republic of Serbia, University of Belgrade, Belgrade, Serbia; ^5^ Department of Musculoskeletal Tissue Regeneration, Bernhard-Heine-Center for Locomotion Research and Orthopedic Hospital König-Ludwig-Haus, University of Würzburg, Würzburg, Germany

**Keywords:** hypophosphatasia, ALPL, TNAP, PDL-hTERT cells, *in vitro*

## Abstract

**Introduction:**

Besides skeletal symptoms, dental abnormalities are a typical feature of the rare inherited disorder hypophosphatasia (HPP), which is caused by loss of function mutations in the *ALPL* gene (alkaline phosphatase, biomineralization associated) coding for tissue-nonspecific alkaline phosphatase (TNAP). Dental symptoms include premature loss of deciduous teeth, disturbance in dentin and cementum mineralization, and an increased risk for periodontitis. However, the underlying molecular mechanisms are not fully understood and experimental cell lines for *in vitro* analyses of these processes are missing.

**Methods:**

We aimed to develop a physiologically relevant cellular model of dental origin with genetic *ALPL* variants to investigate the molecular consequences of TNAP deficiencies *in vitro*. For this purpose, we used immortalized periodontal ligament stem cells (PDL-hTERT cells) to establish five independent clonal cell lines via CRISPR/Cas9, harboring different *ALPL* genetic variants.

**Results:**

Detailed investigation of their genetic properties revealed that four different genotypes were successfully established at two different positions within the *ALPL* gene locus. The detected variants either result in mis-splicing of *ALPL* mRNAs or in frameshift mutations. All determined variants implied severe consequences on TNAP function, as indicated by *in silico* modeling and comparison to reported human pathogenic variants. Subsequent detailed cell culture experiments demonstrated TNAP residual gene expression and altered TNAP activity in the newly established *ALPLtg* PDL-hTERT lines. Further assessment of cell line features showed significantly reduced cell growth, partly lower levels of intracellular ATP as well as mitochondrial function proteins. TNAP activity was furthermore investigated during *in vitro* osteogenic differentiation and strong suppression during this process in nearly all newly established lines was observed.

**Discussion:**

We report the generation of a new set of immortalized *ALPLtg* PDL-hTERT cells for investigation of TNAP cellular function in PDL cells, which can be used in subsequent studies for deciphering molecular processes in dental cells affected by reduction of TNAP function.

## Introduction

### Hypophosphatasia and its clinical classification

Hypophosphatasia (HPP) is a rare, inherited, multisystemic disease manifesting due to mutations in the *ALPL* gene (1p36.12, NCBI-Gene-ID: 249) (Millan and Whyte, 2016). The gene is encoding for the ecto-phosphatase/nucleotidase Tissue-nonspecific Alkaline Phosphatase (TNAP), which is attached to the cellular membrane via a *glycosyl-phosphatidyl-inositol* (GPI)-anchor (Le Du et al., 2001; Garcia et al., 2015; Millan and Whyte, 2016). The prevalence of HPP was estimated to 1:300.000 in severe but to 1:6370 in mild cases in Europe (Mornet et al., 2011). However, a newer published study based on allele classification implies that the occurrence of moderate HPP is 1:2430 and of mild HPP 1:508, which is significantly more frequent than previously assumed (Mornet et al., 2021). Patients suffering from HPP can be classified into several clinical subtypes, depending on the respective age of onset, severity, genetic background and age of diagnosis. Mornet et al. classify the different HPP onsets into the following categories: severe (recessive and rare), moderate (recessive/dominant, more frequent), and mild HPP (dominant and very common) (Mornet et al., 2021). The perinatal and infantile forms lead to a severe onset and are life threatening, whereas other clinical subtypes, like childhood and adult HPP, show a milder onset (Hofmann et al., 2013). Hypomineralization of bones is one of the most prominent consequences of TNAP malfunction in all patients (Millan and Whyte, 2016). Additionally, HPP patients may suffer from problems with muscles, kidneys, and lungs; even neurological symptoms, like depression, anxiety disorders, and epileptic seizures, can occur (Hofmann et al., 2013; Colazo et al., 2019; Bianchi et al., 2020). Interestingly, a rather mild disease subtype only manifesting in the dental system (including alveolar bones) is described as odontohypophosphatasia (odonto-HPP) (Reibel et al., 2009; Hofmann et al., 2013; Martins et al., 2013; Whyte, 2017; Bianchi et al., 2020; Kramer et al., 2021). The high variability of HPP manifestation results in ambiguous observations concerning genotype-phenotype correlations and clinical reports show the actual complexity of the HPP patient´s situation. For example, two siblings carrying the same *ALPL* mutations developed a mild and a severe onset, respectively (Hofmann et al., 2014). Consequently, genetic counseling and impact assessment of detected *ALPL* genetic variants in the context of HPP are still challenging and need to be confirmed by adequate experimental models (Taillandier et al., 2018; Mornet et al., 2021).

### TNAP function during dental mineralization

Common dental phenotypes of HPP patients are a premature loss of deciduous teeth (without resorption of the tooth root), deficits in dentin and enamel, enlarged dental pulps, hypomineralization of the alveolar bones and an increased risk of suffering from inflammatory processes, like periodontitis (Reibel et al., 2009; Whyte, 2017; Kramer et al., 2021). Depending on their genetic status adult HPP patients show a significant higher risk of periodontitis and tooth loss than the general population (Weider et al., 2022). Odonto-HPP can be caused, among others, by mutations in regions of the *ALPL* gene that are coding for the collagen-binding domain (Martins et al., 2013). Generally, TNAP is well-known for its ability to dephosphorylate inorganic pyrophosphate (PPi), which is a central step during most mineralization processes (Harmey et al., 2004). TNAP degrades pyridoxal-5‘-phosphate (PLP), adenosine triphosphate (ATP) step-by-step towards adenosine, lipopolysaccharides (LPL), and phosphorylated osteopontin (p-OPN) (Narisawa et al., 2013; Millan and Whyte, 2016). TNAP is moreover a central player for the propagation of hydroxyapatite (HA) crystallization during bone mineralization as the enzyme diminishes the inhibitor PPi and increases the prevalent concentration of monophosphate Pi, which is a component of HA (Orimo, 2010; Millan, 2013). Several local active enzymes can influence the mineralization process directly by sequestering or reducing TNAP substrates, for example, ENPP1 (Ectonucleotide Pyrophosphatase/Phosphodiesterase 1) (Harmey et al., 2004), ANKH (Inorganic Pyrophosphate Transport Regulator) (Rodrigues et al., 2012) and PHOSPHO1 (Phosphoethanolamine/Phosphocholine Phosphatase 1) (Mohamed et al., 2022). Generally, PPi has a negative effect on the formation of acellular cementum of teeth (Foster et al., 2012). A study published by Kramer et al. describes clear effects of TNAP deficiency on the dental root, the mineralization of mantle dentin, the acellular cementum, Sharpey’s fibers, and the periodontal ligament (PL) of HPP patients (Kramer et al., 2021). The TNAP enzyme is further responsible for decreasing the PPi content and consequently initializing the establishment of acellular cementum at the tooth root (Foster et al., 2012; Zweifler et al., 2015). Application of the TNAP inhibitor levamisole diminished the expression of genes involved in osteogenesis and mineralization as well as the production and turnover of the extracellular matrix (ECM) in human dental pulp and PL stem cells during osteogenic differentiation (oD) (Melms et al., 2020). However, genes involved in inflammatory processes were also expressed at a higher level under levamisole treatment during oD (Melms et al., 2020) and TNAP has been shown to possess an anti-inflammatory role (Komazin et al., 2019), thus might participate in regulation of inflammatory state of periodontitis.

As summarized by Foster et al., *Alpl* knockout mice partly resemble the craniofacial defects of HPP-patients, like defects in mineralizing tissues (cementum, enamel, dentin, and alveolar bones), which can be improved by replacement treatment with the recombinant TNAP enzyme Asfotase alfa (Millan et al., 2008; McKee et al., 2011; Yadav et al., 2012; Foster et al., 2013; Foster et al., 2015). A murine knock-in model, called *Alpl* ^*+/A116T*^ resembling the phenotype of odonto-HPP*,* shows slight TNAP-dependent changes in the acellular cementum as well as in the alveolar bones. The mice did not show any additional skeletal abnormalities, and neither dentin nor enamel defects were displayed, nor did the animals lose their teeth prematurely (Foster et al., 2015). Generally, the impression arises that alveolar bones and teeth may be more susceptible to changes in TNAP functionality than the rest of the skeleton (Foster et al., 2015; Melms et al., 2020).

During normal dental development the periodontal ligament (PDL) is a non-mineralized tissue, essential to connect the tooth cementum to the inner wall of the alveolar bone within the jaw. Moreover, the PDL influences cementum formation, inflammation, tooth mobility and maintenance (Swanson et al., 2022; Wen et al., 2025). The PDL structure contains progenitor cells able to expand and differentiate in different mesenchymal cell linages, including osteoblast-like and fibroblast-like cells (Zeng et al., 2023). PDL cells can be isolated as primary cells, have great differentiation potential and are discussed to be a source for periodontal therapy, they lack prolonged survivability *in vitro* (Swanson et al., 2022). Immortalized PDL cell lines have therefore been generated and are used in cytological investigation, material research and tissue engineering studies. In our study, we used PDL-hTERT cell lines, that is an immortalized periodontal ligament (PDL) progenitor cell line (Docheva et al., 2010). The cell line has been initially generated by using periodontal ligament cells originating from a healthy individual form tooth extraction tissue (age below 25 years, two upper premolars were used as clinical material). After tissue extraction under sterile conditions and initial cell culture growth, immortalization was performed by hTERT Lentiviral transfection at passage one (Docheva et al., 2010). PDL-hTERT cells exhibited, like the primary PDL cells, spindle-like cell morphology which was preserved throughout continuous passaging. In addition, the immortalized cells retain expression of PDL surface markers, e.g., CD73, CD90 and CD105, and retain their differentiation potential. Differences were observed in PDL-hTERT cell growth rates, as these cells divide at a slower rate when compared to PDL primary cells.

The aim of our study was to use immortalized PDL-hTERT cells to model different *ALPL* genetic variants in dental cells. After inducing different mutations in the *ALPL* gene the newly generated cells should be subsequently used to investigate their cellular properties, like differentiation potential and energy metabolism. We hope to gain novel insights into the mechanisms of genotype-phenotype correlations of odonto-HPP by investigating these novel *in vitro* models.

## Materials and methods

### Growth and transfection of PDL-hTERT cells

PDL-hTERT cells were previously generated (Docheva et al., 2010) and were cultured at 37°C and 5% CO_2_ in basal growth medium containing of DMEM High Glucose Medium (DMEM 1x +4.5 g/L D-Glucose, L-Glutamine, - Pyruvate, Gibco, 41965-039) admixed with 10% FCS (Bio-Sell, BS.FCS 0.500 EUA) and 1% Penicillin/Streptomycin (Sigma Aldrich, P4333). To facilitate constant cell growth 5 ng/mL rh FGF (PeproTech, 100-18C–500 µg) was added. Cell media were exchanged every 2 days. Cells were detached at 80%–90% confluency by trypsin digestion (0.05% Trypsin-EDTA in PBS, (Gibco, 15400-54).

Prior to transfection experiments, PDL-hTERT cells were plated at a density of 6250 cells/cm^2^ in growth medium supplemented with 5 ng/mL rh FGF in 6-well plates. Transfection was performed 24 h after seeding at approx. 50% confluent cell layer.

### Generation of *ALPL* ^*tg*^ PDL-hTERT knockout cells

To establish PDL-hTERT *ALPL* knockout cells, a commercial CRISPR/Cas9 system was used (ThermoFisher Scientific). A ribonucleotide transfection solution was established by mixing DMEM high Glucose cell culture medium/1% Pen/Strep (w/o FCS), sgRNA: tracrRNA mix (20 µM), Cas9 protein (5 mg/mL; TrueCutTM Cas9 Protein V2, ThermoFisher Scientific, A36499), and Lipofectamin CRISPRMAX Cas9 transfection Reagent (ThermoFisher Scientific, CMAX00003). Preincubation was performed for 10–15 min at room temperature (RT). Next, cells were incubated with Ribonucleotide transfection solution for 2 days, and subsequently culture medium was replacement daily.

The following crRNAs (TrueGuideTM Synthetic crRNA, ThermoFisher Scientific) were used for transfection.

• ALPL1 construct: *ALPL* transcript ENST00000374840.8/NM_000478.6 exon 6 (ID CRISPR1088188_SGM), target sequence CGTTGTCTGAGTACCAGTCC, PAM: CGG, locus GRCh38 Chr.1:21564117-21564139;
• ALPL2 construct: *ALPL* transcript ENST00000374840.8/NM_000478.6 exon 3 (ID CRISPR1072819_SGM), target sequence CTTGGTCTCGCCAGTACTTG, PAM: GGG, locus GRCh38 Chr.1: 21560639-21560661;
• Positive Control: *CDK4* transcript ENST00000257904.11/NM_000075.4 exon 2 (ID CRISPR1007915_SGM), target sequence TACCTCTCGATATGAGCCAG, PAM TGG, locus GRCh38 Chr.12: 57751690-57751712.

Specific *ALPL* or scrambled control crRNA and tracrRNA (TrueGuideTM Synthetic tracrRNA, ThermoFisher Scientific, A35506) were combined in a premix in TE buffer (pH 8.0), with a final concentration of 20 µM crRNA:tracrRNA duplex in annealing buffer. Heat pretreatment for efficient guideRNAs duplex annealing was performed in a PCR cycler prior to transfection.

A test for efficient Cas9 function on corresponding genomic DNA was performed 2 days after transfection by GeneArt Genomic Cleavage Detection Kit (ThermoFisher Scientific, A24372; Number of cells: 60,000). CRIPSR target locus spanning specific primers (Supplementary Table S1) were used for amplification of target regions. Re-annealing of PCR products and subsequent T7 endonuclease cleavage assays were screened by 1.5% Agarose gel electrophoresis (respective sizes of cleavage products are stated in Supplementary Table S1; Supplementary Figure S1) according to manufacturer’s manuals.

### Monoclonal selection of *ALPL* ^*tg*^ PDL-hTERT cells

Selection of single transfected clones was performed 2 days after transfection. Cells were detached by Trypsin digestion. Subsequently, 100 µL of cell suspension was distributed into single wells within 96-well plates to achieve a final concentration of 0.5 cells/well. Seeded cells were screened 1 day after seeding under light microscope. Wells containing more than 1 cell per well were marked and excluded from further analyses. Wells with single cells were further grown to confluency and stepwise reseeded in well-plates with larger growth area and T75 flasks. Cells were collected for gDNA extraction and genotyping by sequencing after reaching confluent growth. For long term storage, 2 × 10^6^ clonal cells were frozen in media containing 90% FCS and 10% DMSO, and stored at −80°C or −196°C.

### Genetic analyses of novel *ALPL* ^*tg*^ PDL-hTERT clones

All established *ALPL* ^*tg*^ PDL-hTERT single cell-derived clones were investigated via SANGER sequencing and subsequent variant analyses. Genomic DNA was extracted from cell pellets with the NucleoSpin Tissue Kit (Macherey-Nagel, 740952.50) and resuspended in 50 µL elution buffer. *ALPL* gDNA was diluted and used for amplification of corresponding CRISPR targeted regions in *ALPL* exon 3 and 5 via PCR by using primers in the flanking regions (Supplementary Table S1). Successful amplification was controlled via Agarose gel electrophoresis. For subsequent sequencing a clean-up step with ExoSAP-IT (Applied Biosystems, Foster City, United States) was followed by the sequencing reaction using the BigDye Terminator Cycle Sequencing Kit v1.1 (Applied Biosystems, Waltham, United States). Sequencing was conducted on a 3130XL capillary sequencer (Applied Biosystems, Waltham, United States). Sequence analyses were performed by comparison to ENSEMBL *ALPL* reference sequences via SnapGene, CodonCode and ApE software. References to sequences used for amino acid alignments are listed in Supplementary Table S2. Tests for non-specific Cas9 restriction in all clones were performed by gDNA sequencing on computational predicted off-site targets in coding genes (see detailed information in Supplement and Supplementary Table S3) and showed no alterations.

### Immunofluorescence

For immunofluorescence analyses, cells were seeded on glass coverslips to adhere overnight. To label mitochondria, living cells were incubated with pre-warmed growth medium supplemented with 100 nM MitoTracker™ Red CMXRos (#M7512, Thermo Fisher Scientific, excitation wavelengths 579/599 nm) for 30 min at 37°C. The cells were rinsed in PBS with 10% FCS and fixed in 4% paraformaldehyde and stored in the dark at 4°C in 0.1% NaN_3_ (Sigma Aldrich, Israel) in PBS. Next, cells were permeabilized with 0.1% Triton X-100 (Sigma Aldrich, United States) in PBS for 10–15 min at RT and incubated with blocking solution: PBST (0.1% Tween 20 (Sigma Aldrich), 1% bovine serum albumin (BSA, Roche, Germany) in PBS (Sigma Aldrich)) for 30 min at 4°C. Primary mouse anti-human TNAP (B4-78) antibody (#sc-81754, Santa Cruz Biotechnology, United States) was used at dilution 1:100 in PBST overnight at 4°C. After three washing steps in PBS, cells were incubated with secondary goat anti-mouse IgG H&L Alexa Fluor® 488 antibody (#ab150113, Abcam, United States) used at 1:300 dilution in PBST for 1 h at RT in the dark. For the last 10 min, 4′,6-diamidino-2-phenylindole dihydrochloride (DAPI, Sigma Aldrich) was added for DNA staining. Coverslips were mounted with Vectashield Antifade Mounting Medium (Vector Laboratories, United States) onto microscopy slides and stored in the dark at 4°C. For negative controls, cells stained with DAPI or with secondary antibodies only were analyzed. Specimens were checked by fluorescence microscopy (DMi8, Leica Microsystems, Leica Application Suite).

Quantification of TNAP signals in fluorescence images was performed by ImageJ measurements and calculation of corrected total cell fluorescence (CTCF). For this analysis ten random cells per image and five non-overlapping images per cell line were analyzed (n = 50). Subsequently, a Shapiro test showed non-normal distribution and a Kruskal Wallis test was performed for statistical group comparisons.

### Total cell lysate and mitochondria-enriched fraction: extraction and protein concentration measurements

Total cell lysates were collected for each time point (e.g., after 0, 3, and 7 days) and condition from 3 wells seeded with 200,000 cells in a 6-well plate. Cells were washed twice in PBS with protease inhibitor (Protease Inhibitor Cocktail Tablets, Roche 04693116001) and then mechanically scratched from the wells. All wells from one experimental time point and condition were pooled and homogenized. Samples were subsequently centrifugated (5 min, 3000 rpm, 4°C), the supernatant was discarded, and the pellet was eluted in 350 µL PBS with protease inhibitor. Subsequently, cells were sonicated (80% intensity, 10 pulses), centrifugated and the supernatant was collected for further experiments.

For preparation of mitochondria-enriched fraction (MEF), cells were seeded in T75 flasks and upon reaching 80%-90% confluency, cells were collected by scraping in freshly prepared MEF buffer containing 20 mM Hepes (pH 7.6), 220 mM mannitol, 70 mM sucrose, 1 mM EDTA, 0.5 mM PMSF, 2 mg/mL BSA for 20 min on ice. Lysates were homogenized by 1 mL syringe with a 26 G needle and centrifuged at 800 × *g* for 5 min at 4°C. Supernatant containing mitochondria was centrifuged at 10,000 × *g* for 10 min at 4°C. Supernatant was removed, and the resulting pellet (crude mitochondria) was resuspended in MEF buffer without BSA.

Protein concentration was determined with Pierce BCA Protein-Assay-Kit (ThermoFisher Scientific, 23225). Cell lysates and MEFs were denatured and reduced using 5× Laemmli Buffer with 2-mercaptoethanol at 95°C for 5 min. Specifically, MEFs were dissolved in non-reducing 5× Laemmli Buffer for analyses of mitochondrial respiratory chain complexes.

### Western blot analyses

10 μg of total protein was loaded on 10% acrylamide gels followed by sodium dodecyl sulfate polyacrylamide gel electrophoresis (SDS-PAGE). Cell lysate proteins were transferred to the 0.22 μm pore size nitrocellulose membrane (GE Healthcare) while MEF proteins were transferred to the 0.2 μm pore size PVDF membrane (Carl ROTH). Membranes were blocked with 5% BSA (Roche) or non-fat dry milk (Carl ROTH) in Tris (Applichem)-buffered saline with 0.1% Tween-20 (Sigma-Aldrich) and then incubated with primary antibodies directed against human epitopes: mouse anti-TNAP (#MAB29092, R&D), mouse anti-β-actin (#3700, Cell Signaling Technology), rabbit anti-HSP60 (#12165, Cell Signaling Technology), rabbit anti-TOM20 (# ab186735, Abcam), and Total OXPHOS Human WB Antibody Cocktail (#ab110411, Abcam) (all at a dilution of 1:1000) overnight at 4°C. Afterwards, membranes were either incubated with an anti-rabbit or anti-mouse immunoglobulin G (IgG) antibody conjugated with horseradish peroxidase (both diluted 1:1000, Cell Signaling Technology). Protein bands were visualized by using WesternSure® PREMIUM Chemiluminescent Substrate (Licor) and images acquired using the FluorChem Q Imaging System (Cell Biosciences) or C-DiGit® Blot Scanner (Licor).

### *In vitro* osteogenic differentiation

For long time *in vitro* differentiation experiments of PDL-hTERT, cells were grown on fibronectin (10 μg/mL in PBS, Promocell, C43060) coated 6- or 24-well plates to prevent detachment of cells during the treatment. Cells were initially seeded at 20,000 cells/cm^3^ density in triplicates without rh FGF supplementation until confluency. Basal growth medium was exchanged with differentiation medium (basal medium substituted with 10 mM β-Glycerophoshate (Sigma-Aldrich, G9422), 100 nM 2-Phospho-L-ascorbic acid trisodium salt (Sigma-Aldrich, 49752) and 100 nM Dexamethasone (Sigma-Aldrich, D4902). Control cells were seeded in comparative densities and grown with basal growth medium without additives. Cells were differentiated up to 28 days, depending on the follow-up experiment. Details on PTH stimulation are given in the Supplementary Material.

### RNA isolation and cDNA synthesis

For analyses of gene expression, RNA was extracted from pooled cells at different time points of osteogenic differentiation (1, 3, 7 and 14 days, initial seeding density 200,000 seeded cells/well). Cell medium was discarded, and cells were initially washed with sterile PBS buffer prior to lysis. Cells were mechanically detached by scraper, and RNA extraction was performed with the NucleoSpin RNA II Kit (Macherey-Nagel, NZ740955250). The total RNA was diluted in 20 µL nuclease-free water and stored at −80°C. RNA concentration was photometrically determined.

cDNA synthesis was performed with 1 µg RNA per sample with M-MLV reverse Transcriptase (Promega, M170B) according to the manufacturer protocol in the corresponding M-MLV Reverse Transcriptase 5x Reaction Buffer (Promega, M531A) and by utilizing Oligo-dT Primer (50 pmol/μL, Promega, C110A). Samples mixed with Oligo-dT Primer were 70°C heat pretreated for 5 min cDNA synthesis including all components was performed on a PCR cycler for 1 h at 42°C with subsequent enzyme heat deactivation.

### Gene expression analyses by quantitative real-time PCR (qPCR)

Quantification of gene expression was conducted by qPCR measurements. cDNAs of different samples were diluted 1:10 and 2 µL were used per qPCR reaction. GoTaq® qPCR Master mix 2x (Promega, A6002) was used as premixed enzyme solution along with transcript-specific primers (detailed primer information is listed in Supplementary Table S1). qPCR reaction was performed on a C1000Thermal Cycler with a CFX96 touch Real-time PCR detection system (Bio-Rad) and analyzed with Bio-Rad CFX manager. Relative expression values were calculated via the 2^−ΔΔCT^ method and normalized to beta-2-microglobulin (B2M) expression levels.

### Intracellular ATP content measurement

ATP levels were quantified using the CellTiter-Glo® Luminescent Cell Viability Assay (Promega, #G7570) in accordance with the manufacturer’s recommendations. A total of 1,000 cells/well were used in opaque 384-well plate (Greiner). Luminescence, directly proportional to the concentration of ATP was detected and ATP content was determined based on standard curve prepared using ATP disodium salt (Sigma Aldrich, # A7699).

### TNAP activity measurement - CSPD assay

TNAP protein activity was quantified by measuring chemiluminescence levels after CSPD substrate degradation in cell lysates. Protein lysates were extracted from cultured cells (3 wells of a 24-well plate for each condition) and for each well 10 µg total protein was diluted in PBS buffer. 100 μL sample solution was mixed with 100 µL Ready to use CSPD Reagent (Merck, 11755633001) and incubated for 5 min at 37°C prior to measurement. Luminescence was quantified with Tecan Infinitive plate reader (duplicate measurements, white 96-well plate with transparent bottom). Levamisole hydrochloride (Merck 31742, final concentration 1 mM) was added to suppress phosphatase activity of non-*ALPL* isoforms in the corresponding samples of the CSPD assay.

### TNAP activity staining - naphthol/fast blue assay

Cells were investigated after 7 und 14 days of differentiation (3 wells of a 24-well plate for each condition) for their biochemical activity of alkaline phosphatases, which was visualized by the Naphthol/Fast Blue reaction. The Leukocyte Alkaline Phosphatase Kit (Sigma Aldrich, 86C-1 KT) was used with slight adaptation to monolayer cells grown in 24-well plates. In detail, the corresponding cell growth medium was removed, cells were washed twice with PBS and fixed in 4% paraformaldehyde (Morphisto, 11762.01000) for 30 min at RT. After additional deionized water washing steps, the samples were incubated with staining solution (0.6 mg/mL Fast Blue RR (Sigma Aldrich, F0500), 0.2 mg/mL Naphthol AS-MX Phosphate disodium (Sigma Aldrich, N5000) in 0.1 mol/L Tris puffer (pH 9.0)) on a shaker for 15 min at RT and subsequently washed three times with deionized water. Lastly, stained cells were analyzed with a Leica DMi8 microscope (Leica Microsystems), and color images were acquired to visualize blue labelled substrates in alkaline phosphate positive cells.

### Statistical analyses

Statistical analyses were performed via GraphPad Prism (version 8). Shapiro-Wilk test was used to test for normal distribution of values. Analyses of variance in normal distributed groups were performed by one-way ANOVA with Dunnett correction. Non-parametric test was performed with Kruskal–Wallis test including Dunn´s Post-hoc test. Two independent samples groups were analyzed by unpaired t-test and corrected for multiple analyses with calculations according to Holm-Sidak. The level of significance was set to 0.05.

### Used software

Alamut™ Visual Plus (Version 2.15; Sophia Genetics; Lausanne, SW and Boston, United States; https://www.sophiagenetics.com/platform/alamut-visual-plus/)
MEGA 11, MUSCLE algorithm for inter-species alignment of aa sequences, given positions are human aa residues; https://www.megasoftware.net/)
ApE (by Wayne Davis; https://jorgensen.biology.utah.edu/wayned/ape/)
SnapGene software (Dotmatics; www.snapgene.com)
CodonCode Aligner (CodonCode Corporation, https://www.codoncode.com/aligner/)

### Open-access databases

ENSEMBL.org (July 2024, http://www.ensembl.org/index.html)
PubMed and NCBI (June 2024, https://pubmed.ncbi.nlm.nih.gov/)
AlphaFold and AlphaFold 3 (July to Sep. 2024, https://alphafold.ebi.ac.uk/)
UniProt (Sep. 2022, https://www.uniprot.org/)
Swiss-Model and Expasy web server (July 2024, https://swissmodel.expasy.org/)
ALPL gene variant database (June 2024, https://alplmutationdatabase.jku.at/)

## Results

### Generation of *ALPL* ^*tg*^ PDL-hTERT single cell-derived clones

**FIGURE 1:**
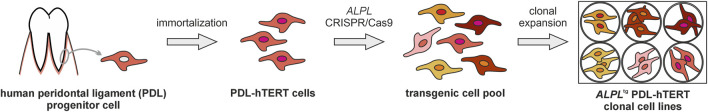
Schematic experimental overview of *ALPL* ^*tg*^ PDL-hTERT cell line generation. A PDL-hTERT cell line derived from primary human PDL progenitor cells (Docheva et al., 2010) was used to generate cell lines with mutations in the *ALPL* gene. CRISPR/Cas9 genome editing vectors with two different guide RNAs that were specific to regions in exon 3 and 6 were applied, respectively. After transfection of bulk PDL-hTERT cells, single cell clones were obtained by limited dilution and subsequently expanded. The genotype of *ALPL* ^*tg*^ PDL-TERT cell lines was evaluated by Sanger sequencing and five transgenic (tg) cell lines and one transfected, but *ALPL* genetically unaltered cell line (wt) were selected for further analyses.

Establishment of *ALPL* ^*tg*^ PDL-hTERT single cell-derived cell lines was performed via gRNA and CRISPR transfection targeting exon 3 and exon 6 of the *ALPL* gene (Figure 1). The exons were selected, as they are partly coding for the alkaline phosphatase protein domain and genetic variations should result in altered enzymatic TNAP function. Initial cell transfection and cleavage tests at the corresponding genomic DNA loci were performed and indicated successful genome editing at the *ALPL* locus with sufficient efficiency (Supplementary Figure S1). Four transfection replicates showed a mean efficiency of 12.73% (SD = 5.00%) for ALPL1 and 15.97% (SD = 6.95%) for ALPL2 sgRNA transfection. The efficiency for a single CDK4 sgRNA transfection as positive control was calculated to 25.88%.

Separation of successfully transfected cells and their clonal expansion led to the establishment of initially 22 growing clonal colonies for each *ALPL* scRNA construct. Subsequent sequencing and screening for induced variations in the *ALPL* genetic locus reduced this number down to eight genetically different *ALPL1* clonal lines (36.36%) and three genetically different *ALPL2* clonal lines (13.64%). Five different *ALPL* ^*tg*^ PDL-hTERT clone lines were selected due to cellular characteristics and genetic properties for further molecular analyses (clones 1.1, 1.2, 1.3, 1.5 = ALPL1 sgRNA/exon 6, and 2.3 = ALPL2 sgRNA/exon 3). Moreover, one not transfected (PDL-hTERT) and one *ALPL2* sgRNA transfected, but genetically unaltered PDL-hTERT clone line (wt) were included as controls in further experiments.

### Genetic analyses of novel *ALPL* ^*tg*^ PDL-hTERT clones

**FIGURE 2:**
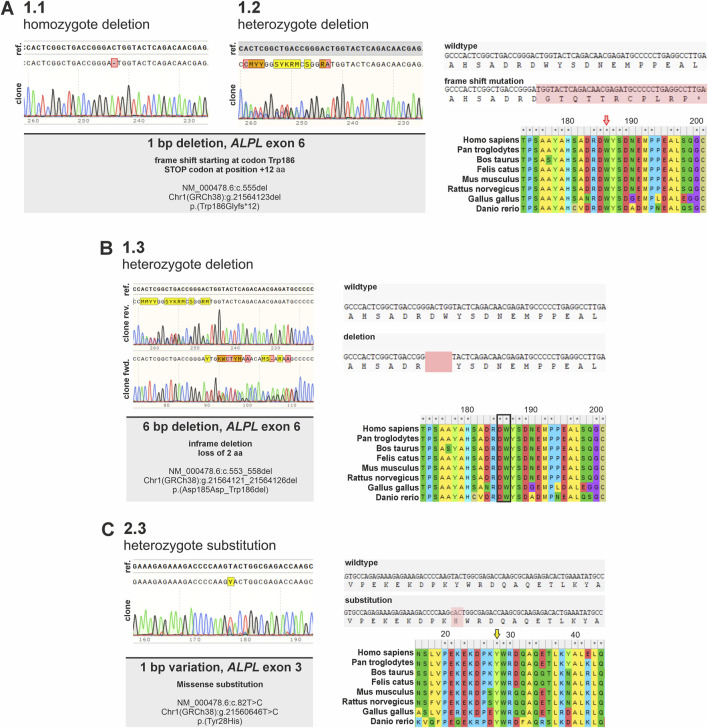
Detection of genetic consequences of *ALPL* variants identified in different *ALPL* ^*tg*^ PDL-hTERT clonal cell lines. Sanger sequencing results of genomic DNA of different *ALPL* ^*tg*^ PDL-hTERT clones within the targeted *ALPL* locus validating different genetic alterations in the lines are shown. **(A)** Sequencing of gDNA from clone 1.1. and 1.2. indicate in both clones a 1 bp deletion in *ALPL* exon 6, resulting in a frameshift in the corresponding coding sequence at aa position 189 (marked in red) and a predicted premature stop codon after additional 12 aa (marked by an asterisk). Both clones differ, as clone 1.1. is homozygote, while clone 1.2 is heterozygote. **(B)** Sequencing of gDNA from clone 1.3 indicates a heterozygote variant, which results in a 6 bp in-frame deletion. This variant leads to a loss of two evolutionary conserved amino acid residues. **(C)** Clone 2.3 displays a 1 bp heterozygote substitution. This variant results in an aa change at the evolutionary highly conserved Tyrosine 28 with potential pathogenic consequences (Alpha Missense Pathogenicity score: 0.615). Alignments show evolutionary conservation of affected regions in different vertebrate species.

**FIGURE 3:**
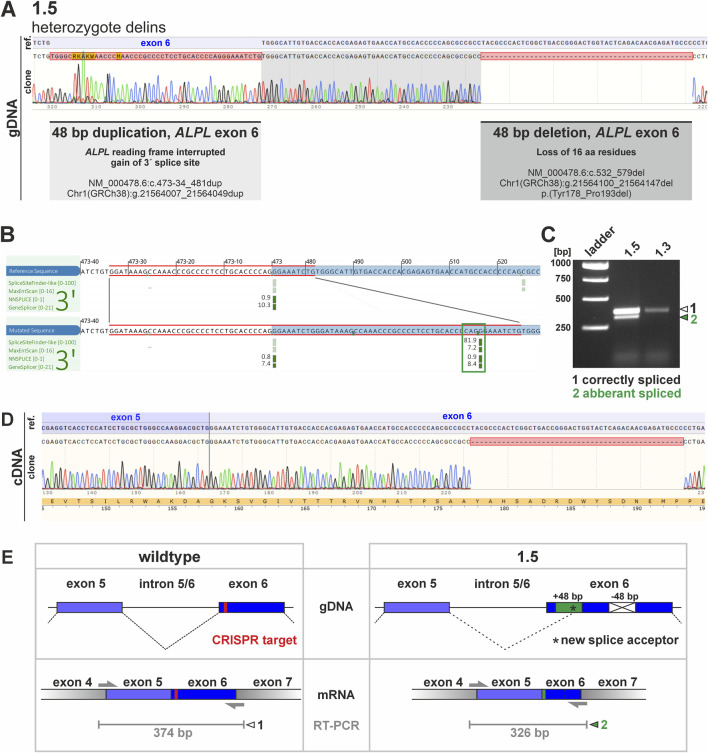
Potential consequences of genetic changes in clone 1.5. and investigation of mRNA products via RT-PCR. **(A)** Sequencing of clone 1.5 gDNA showed a huge genetic alteration within the *ALPL* exon 6 locus, including a 48 bp duplication and a 48 bp deletion. Clonal cDNA sequencing further clarified that the deletion results in an in-frame loss of 48 bp, thereby subsequently should result in loss of 16 aa within the corresponding TNAP protein domain. The corresponding insertion is not detected in the cDNA sequencing. **(B)** A potential gain of a novel splice acceptor site was predicted by different algorithms (Alamut Visual Plus splice summary is shown, blue box marks *ALPL* exon 6 locus, red box indicates gained sequence, green box marks novel splice site). **(C)** The *in silico* predicted novel splice product was detectable via RT-PCR by electrophoresis. The analyses indicate an additional PCR product in clone 1.5. While the larger product (marked with white arrowhead) is also present in clone 1.3, the smaller band was only detected in clone 1.5 (marked with green arrowhead). **(D)** Sequencing of the smaller RT-PCR products shows deletion of 48 bp and the active usage of the new splice site. **(E)** Schematic presentation of sequence aberration and splice prediction for the wildtype and the aberrant sequence on gDNA (upper panel) and mRNA level (lower panel). PCR primer binding sites and expected RT-PCR product sites (in grey) are in addition sketched below. The CRISPR target region is marked in red. The green box marks 48 bp duplication in clone 1.5, while the crossed white box indicates deletion of 48 bp. Black asterisk marks position of potential novel splice site.

For validation of genetic changes, gDNA and cDNA from all lines were extracted, the targeted *ALPL* locus was amplified by PCR, sequenced via Sanger-Sequencing and subsequently aligned to a reference sequence (NM_000478.6). Table 1 and Figures 2, 3 summarizes detected changes in five different *ALPL* ^*tg*^ PDL-hTERT clones and implies potential consequences on the mRNA level and subsequent amino acid (aa) changes within the TNAP protein. Tests for non-specific Cas9 restriction in all clones were performed by gDNA sequencing on CRISPOR predicted off-site targets in coding genes (detailed information in Supplement, Supplementary Table S3) and showed no additional genetic alterations.

**TABLE 1:** Validated genomic alterations in *ALPL* ^*tg*^ PDL-hTERT clone**s**. Potential consequences (gDNA, cDNA and protein alterations; homo; homozygous variant; het: heterozygous variant) and similar known variants (as reported on *The ALPL gene variant database*). All detected variants affect the AP-like core domain of TNAP (IPR001952). Predicted severity of the clinical pathology, which might potentially be caused by different alleles, is according to Mornet et al. (2021); s (severe recessive), m (moderate), N (undetermined), N.A. (not available).

Clone name	gDNA variant ENSEMBL Chr1 (GRCh38)	cDNA transcript variant NM_000478.6	Allele	Protein variant UNIProt P05186	Potential consequences	Predicted severity	Previous reported, similar variants
1.1	g.21564123del	c.555del	homo	p. (Trp186Glyfs*12)	Frameshift, premature STOP codon after additional 12 aa same variant as clone 1.2	s/s: very severe or lethal s/N: mild	c.558G>A; p. (Trp186*) nonsense, Pathogenic Variation ID: 1073912 Accession: VCV001073912.7 (Garcia-Fontana et al., 2019; Mornet et al., 2021)
1.2	g.21564123del	c.555del	het	p. (Trp186Glyfs*12)	Frameshift, premature STOP codon after additional 12 aa same variant as clone 1.1	s/s: very severe or lethal s/N: mild	c.558G>A; p. (Trp186*) nonsense, Pathogenic Variation ID: 1073912 Accession: VCV001073912.7 (Garcia-Fontana et al., 2019; Mornet et al., 2021)
1.3	g.2154121_21564126del	c.553_558del	het	p. (Asp185_Trp186del)	In frame deletion of 2 aa	m/m: severe, non-lethal m/N: mild	none
1.5	g.21564055_21564145delins90	c.487_577delins90	het	p. (Tyr178_Pro193del)	In frame deletion of 16 aa	s/N: mild	c.532T>C; p. (Tyr178His) missense, Pathogenic Variation ID: 1076160 Accession: VCV001076160.12 (Garcia-Fontana et al., 2019; Li et al., 2020) c.565_575delinAG p. (Asp189_Met192delinsArg) The ALPL Gene VariantDatabase Dr I. Rau, Jan. 2019
2.3	g.21560646T>C	c.82T>C	het	p. (Tyr28His)	Missense variant (TAC to CAC)	N.A.	c.82T>G; p. (Tyr28Asp) Odonto HPP, Pathogenic Yang et al. (2013), Liu et al. (2021) c.83A>G; p. (Tyr28Cys) Infantile HPP, Pathogenic Taillandier et al. (2001)

Sequences obtained from clone 1.1 and 1.2 indicated a 1 bp deletion in *ALPL* exon 6 in both clones. This variation results in a frameshift starting at aa 186 within the corresponding protein sequence and is predicted to result in a premature stop codon after additional 12 aa. Both clones genetically differ, as clone 1.1. carries the homozygous variant, while clone 1.2 carries a heterozygous variant (Figure 2A). Clone 1.3 gDNA and cDNA clonal sequencing indicates a heterozygous variant in *ALPL* exon 6 of this cell line, which results in a 6 bp in-frame deletion. This variant is predicted to result in loss of two evolutionary conserved amino acid residues (Figure 2B) and implies severe functional consequences. Clone 2.3 displays a 1 bp heterozygous missense substitution in *ALPL* exon 3. This variant results in an aa change at the evolutionary highly conserved Tyrosine 28 with potential pathogenic consequences (Alpha Missense Pathogenicity score: 0.615). Genomic DNA sequencing of clone 1.5 implies a heterozygous delins variant in the *ALPL* exon 6 locus (Figure 3). Besides the duplication of 48 bp, that are spanning the splice acceptor of exon 6, an additional 48 bp deletion is detected 50 bp further downstream within exon 6 (Figure 3A). Subsequent, cDNA sequencing validates the deletion also at the mRNA level and implies functional consequences on the TNAP protein, but did not show the sequence duplication in exon 6 (Figure 3A). Prediction of splice site consequences after the 48 bp duplication via Alamut Visual Plus software (combining four different splice algorithms) resulted in identification of a high-scoring novel splice acceptor site in this region (Figure 3B; SpliceSite Finder score: 81.9; MaxEntScan score: 7.2; NNSPLICE score: 0.9; GeneSplicer score: 8.4). RT-PCR experiments further validated that clone 1.5 expresses two different *ALPL* transcripts, which display clear size alterations (Figure 3C) corresponding to the 48 bp deletion. According to the cDNA sequencing data, the gain of the new splice site and the aberrant splicing results in the wildtype sequence of *ALPL* exon 6, except of the deleted region (Figure 3D). The different sequencing results propose a model sketched in Figure 3E, which indicates the in-frame loss of 18 aa in this variant and suggests mild consequences according to published database recordings (Table 1).

### TNAP expression and residual activity in *ALPL* ^*tg*^ PDL-hTERT lines

**FIGURE 4:**
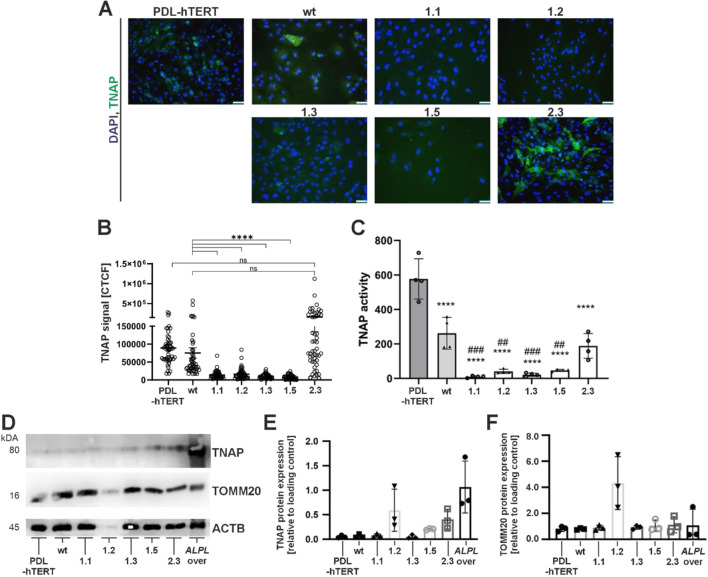
Analyses of TNAP expression and activity by immunofluorescence staining, Western blot, and enzyme activity assay. Cell analyses were performed in five different *ALPL* ^*tg*^ PDL-hTERT cell lines, the wt line and control PDL-hTERT that did not undergo genome editing. **(A)** TNAP expression (green) was detected by immunofluorescence after nuclear counterstaining with DAPI (blue). Representative images are shown. Scale bars = 50 µm. **(B)** Quantification of TNAP signals in IF images were done by corrected total cell fluorescence (CTCF) for ten random cells per image, and five images per cell line (n = 50). **(C)** Specific TNAP activity was measured by a CSPD assay confirming the spontaneous activity of the expressed protein. N = 4 per condition. **(D–F)** TNAP expression level and the expression of the mitochondrial protein TOMM20 in total cell lysates were determined semi-quantitatively by Western blot analysis in relation to the housekeeping protein β-actin/ACTB. A lysate of a TNAP overexpressing cell line (*ALPL* over) was used as positive control. A representative immunoblot is shown in **(C)**. White pixels indicate signal oversaturation. Quantification results in E and F are presented as mean ± SEM, N = 3 per condition.

To investigate *ALPL* expression and residual activity in the new *ALPL* ^*tg*^ PDL-hTERT lines, we performed immunofluorescence analyses (IF; Figure 4A, IF quantification by corrected total cell fluorescence; Figure 4B), quantified residual TNAP activity by CSPD assays (Figure 4C), and performed protein detection by Western blot (Figure 4D–F). Clones 1.1, 1.2, 1.3 and 1.5 showed low TNAP overall expression in IF experiments and low levels of residual TNAP activity. Clone 1.2 displayed rather weak protein bands and showed very low TNAP and beta-actin levels (Figure 4D). Low protein levels in this clone were repeatedly detected during independent experiments. The corresponding relative protein level quantification thereby imply high levels of TOMM20 protein (Figure 4F). In comparison to other clones, our results showed increased cellular TNAP protein expression in clone 2.3 (Figures 4A,B,D,E), which correlated with a clearly detectable TNAP activity in this clone (Figure 4C). Although PDL-hTERT and non-transfected cells (wt) did not show strong protein TNAP expression (Figures 4A,E), their TNAP activity was higher than in other cell lines (Figure 4C).

### Phenotype investigation of *ALPL* ^*tg*^ PDL-hTERT lines during proliferation

**FIGURE 5:**
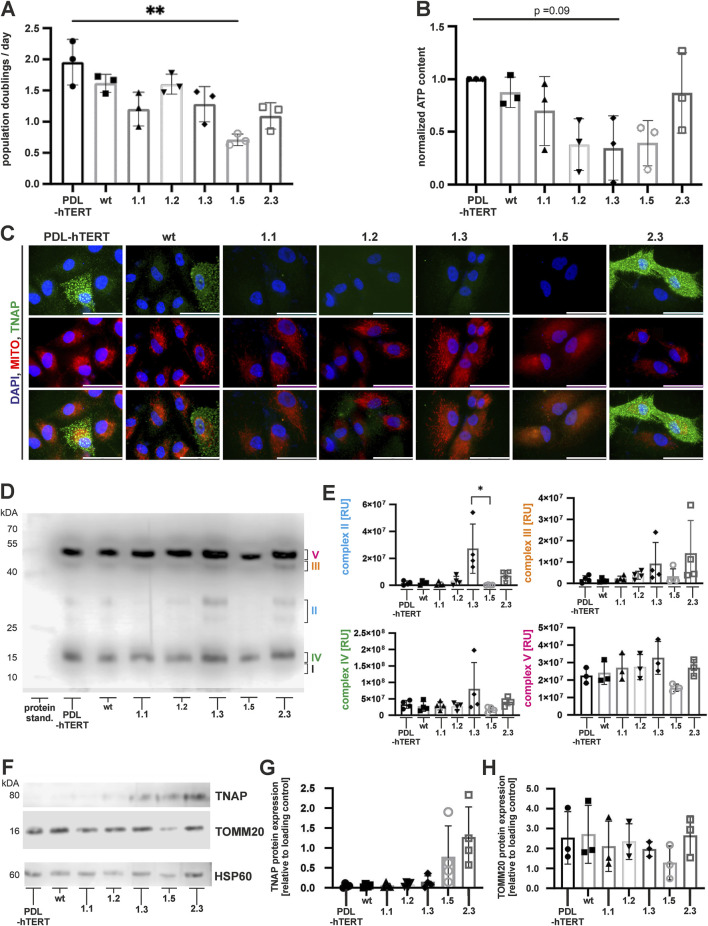
Cell proliferation, metabolic activity, and expression of mitochondrial TNAP. Population doubling time **(A)** during expansion culture and the intracellular ATP content **(B)** were determined at three independent culture time points of each cell line. **(C)** Representative immunofluorescence microscopy images of TNAP (green) and mitochondria (MitoTracker-Red; red), following nuclear counterstaining with DAPI (blue). Scale bars = 50 µm. **(D)** A representative immunoblot of non-reduced cell-derived mitochondria-enriched fraction (MEFs) analyzed for the expression of mitochondrial proteins of the respiratory chain: Complex I (subunit NDUFB8), Complex II, Complex III, Complex IV, and Complex V (ATP synthase subunit alpha). **(E)** Semi quantitative analysis of MEF proteins. N = 4 per condition **(F)** Representative immunoblots of reduced MEFs for mitochondrial TNAP and TOMM20. Quantification results of TNAP **(G)** and TOMM20 **(H)** are depicted as fold changes to loading control (HSP60). Results are presented as mean ± SEM, N = 3 per condition.

Next, we investigated the cell growth, ATP production, and mitochondrial functions in the newly established lines. Our results showed a significantly low population doubling in clone 1.5 (Figure 5A, details on cell number determination and proliferation assay are given as a Supplementary Material), which was in line with low ATP production in this clone (Figure 5B). Low normalized ATP content was also observed in clone line 1.2 and 1.3 (Figure 5B). However, both lines displayed only slightly reduced population doubling rates (Figure 5A). Additional investigation of TNAP protein expression together with mitochondria staining (MitoTracker-red) was performed by immunofluorescence (Figure 5C). TNAP protein was detected in control, wt and clone 2.3. cells, while all other lines did not show any residual TNAP protein as previously observed (Figure 4A).

Interestingly, we could not observe significant relations between cell growth, ATP production, and expression of molecule enabling protein-transporting ATPase activity (TOMM20) in total cell lysates (Figures 4C,E). Similarly, we did not observe clear colocalization between TNAP and mitochondria in PDL-hTERT cells (Figure 5C). To decipher a potential link between altered TNAP and mitochondrial function, we extracted electron transport chain complexes and quantified the protein composition in mitochondria-enriched fractions (MEFs) by immunoblotting (Figures 5D,E). However, when analyzing protein extracts of *ALPL* ^*tg*^ PDL-hTERT cells, we observed significantly lower expression of complex II unit (succinate dehydrogenase (SDH)) in clone 1.5, compared to clone 1.3 (Figures 5D,E). Importantly, we found clone 1.5 lacked four identified mitochondrial electron transport chain complexes (Figure 5E), which might relate to their low population doubling and ATP production. Interestingly, there was a tendency of higher expression of mitochondrial electron transport chain complexes in clone 1.3. To check for a link between mitochondrial function proteins and cell growth, we analyzed the expression of TNAP in the MEFs isolated from *ALPL* ^*tg*^ PDL-hTERT, which revealed a slightly stronger TNAP expression in mitochondria derived from clones 1.5 and 2.3 (Figures 5F,G). Taken together, our results showed the strongest TNAP protein expression in cell lysates and mitochondrial fraction of clone 2.3, which correlated with strongest spontaneous TNAP activity when compared to other clones but not to PDL-hTERT or wt cells.

### Phenotype investigation of *ALPL* ^*tg*^ PDL-hTERT lines during osteogenic differentiation

**FIGURE 6:**
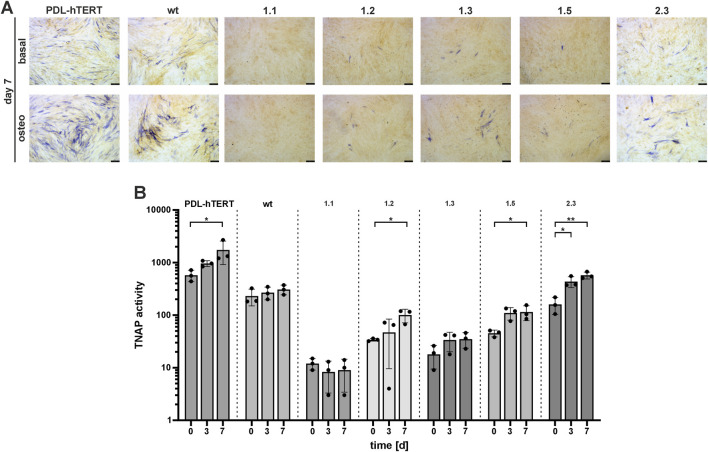
TNAP activity during *in vitro* osteogenic differentiation. Cell lines were cultured in osteogenic induction medium (DMEM high glucose medium, 10% FCS, basal medium substituted with 10 mM β-Glycerophoshate, 100 nM L-Magnesium Ascorbyl Phosphate, and 100 nM Dexamethasone) and **(A)** TNAP was detected by staining with a colorimetric substrate after 7 days of incubation. Representative microscopy images are shown. Scale bars = 50 µm. **(B)** Specific TNAP enzyme activity was assessed at three independent time points (day 0, 3, and 7) using a CSPD assay and the AP-specific inhibitor levamisole. Results are presented as mean ± SEM, N = 3 per time point, log10 scale.

PDL-hTERT cells show potential to differentiate into osteogenic cells, after prolonged incubation in osteogenic induction medium (investigation of marker gene expression in PDL-hTERT cells during osteogenic differentiation is shown in Supplementary Figure S6) (Docheva et al., 2010). To test the potential of the *ALPL* ^*tg*^ PDL-hTERT derived cells, we investigated TNAP function in these cells after 7 days of differentiation by Naphthol/Fast Blue assay (Figure 6A). Cells grown in basal medium displayed only a low amount of blue stained TNAP-positive cells and thereby showed only a low level of spontaneous differentiation potential (Figure 6A, upper panel). Differentiation of cells resulted in raised levels of differentiation potential of control cell lines (PDL-hTERT and wt) and in clone 2.3 cells. Low number of Naphthol/Fast Blue positive cells were detected in lines 1.1, 1.2, 1.3, and 1.5 (Figure 6A, lower panel). The corresponding quantification of TNAP activity by using a CSPD assay and the AP-specific inhibitor levamisole, further showed that cell lines with rather normal differentiation potential significantly increased their TNAP activity over time (Figure 6B). In accordance with the rather weakly stained Naphthol/Fast Blue assay, this assay also showed that cell lines 1.1, 1.2, 1.3, and 1.5 have only low residual TNAP activity. Their TNAP activity can partly increase after incubation in differentiation medium but remained at low levels compared to control cells. In contrast, line 2.3 showed significantly higher levels of TNAP activity after 7 days of differentiation and reinforced the observed functional divergences observed in this line. Collectively, our results showed the strongest TNAP protein expression in cell lysates and mitochondrial fraction of clone 2.3 (Figures 4, 5). This correlated with strongest TNAP activity induced by osteogenic stimuli when compared to other clones but not to PDL or wt cells.

## Discussion

The aim of this study was to establish and characterize a set of novel odontogenic cell lines harboring different genetic alterations in the *ALPL* gene. *ALPL* encodes for the TNAP enzyme, which is essential for regulating cellular phosphate levels and for the correct establishment of calcified structures, like bones and teeth, during development in vertebrates (Millan and Whyte, 2016; Mornet, 2018). Patients lacking TNAP function develop different kinds of hypophosphatasia, a rare inherited disease with variable phenotypes, ranging from severe, prenatal lethal to rather mild, adult forms (Mornet et al., 2021). One special HPP phenotype is odonto-HPP (OMIM # 146300), as it only affects dental tissues and results prominently in premature loss of primary teeth and tooth malformations (Melms et al., 2020; Wölfel et al., 2023). Intriguingly, preterm lost teeth do not resorb tooth roots and lack any acellular cementum (van den Bos et al., 2005; Zweifler et al., 2015), a mixture of inorganic material/hydroxyapatite and water but lacking organic material/collagens. The acellular cementum is a nonmineralized, supporting tooth structure covering large parts of the tooth root and is essential for periodontal ligament (PDL) attachment and tooth stability. Although the genetic and biochemical function ALPL/TNAP have been investigated for many years in a wide number of different cell culture and animal models (Foster et al., 2013; Foster et al., 2015; Graser et al., 2015; Williams et al., 2018; Ohlebusch et al., 2020), rather few investigations have yet focused on modeling specific dental features after TNAP depletion *in vitro* (Tomlinson et al., 2015). Until today, a steady growing number of different immortalized dental cell lines have been reported and are available for experimentation (Zeng et al., 2023). We used the previously established PDL-hTERT cell line, which represents an immortalized cell line that can be osteogenically differentiated *in vitro*, including calcified structures (Docheva et al., 2010). PDL-hTERT cells have been shown to be able to grow on different titanium surfaces, to have stable proliferation potential and resemble a primary PDL cell like phenotype.

### Cell line mutations in *ALPL* ^*tg*^ PDL-hTERT cells and potential functional consequences

We targeted two different genetic loci within the *ALPL* gene (exon 3 and 6) to be altered via CRIPSR/Cas9 transfection to interfere with TNAP function in PDL-hTERT cells. Subsequent, clonal selection resulted in detection of overall 44 clones (22 growing clonal colonies for each *ALPL* scRNA construct) resembling 12 distinct *ALPL* variants. Five different clones were selected due to their genetic mutation, cellular growth properties and exclusion of potential off-target Cas9 activity (Supplementary Figures S2, S3).

Clones 1.1 and 1.2 depict the same 1 bp deletion variant, which is predicted to result in a frameshift within the *ALPL* coding sequence and to subsequently interfere with TNAP function. Translation of the c.555del transcript variant in a p. (Trp186Glyfs*12) protein should result in gain of 12 novel amino acids and a truncated protein due to a premature stop codon. Current literature and comparison to previously reported similar genetic variants indicate potential severe consequences and report up to pathogenic HPP forms, e.g., c.558G>A; p. (Trp186*) (Garcia-Fontana et al., 2019; Mornet et al., 2021). Other reported *ALPL* deletion variants in proximity to this region are known to induce severe pathogenic HPP in compound-heterozygote backgrounds, e.g., c.427del; p. (Gln143Argfs*22) (Whyte et al., 2015), and c.544del; p. (Ala182Leufs*16) (Taillandier et al., 1999). Prediction of 3D protein structure by AlphaFold 3 (Abramson et al., 2024) implies strong alterations in the corresponding TNAP dimer formation (Supplementary Figure S5B).

Clone 1.3 displays a heterozygote 6 bp deletion in *ALPL* exon 6 and hints to enzymatic reduction, as the two affected amino acid residues are evolutionary highly conserved and are predicted to significantly change protein 3D structure (Supplementary Figure S5D). However, the variant should not cause a full loss-of-function, as one chromosomal copy still functional and thereby could still compensate TNAP function. Genetic position in exon 6 of *ALPL* is similar to the variant detected in clone 1.1. and 1.2, as same crRNA has been used to generate this line. Deletion variants in this region have not been reported in HPP patients, yet. Nevertheless, a genetic variant at this position has been recently published by direct submission to *ALPL* Gene Variant Database (Farman et al., 2024). The c.565_575delinsAG, p. (Asp189_Met192delinsArg) variant is not clinically defined, but is linked to an adult-onset HPP patient.

The genomic alterations detected in clone 1.5 indicate rather one indel event, or two independent changes in this line within exon 6 of *ALPL*. First, an insertion of a 48 bp sequence, which corresponds to the 5′ downstream sequence directly adjacent to the duplication site. The insertion results in gain of an active, additional splice site, which is predicted by ALAMUT Software according to MaxEntScan, MNSPLICE, and GeneSplicer (Figure 3). This variation cannot be detected on mRNA level, as the new splice product does not alter sequence or size of the corresponding mRNA transcript. Second, the genetic changes resulted in deletion of 48 bp in exon 6 and is predicted to result in an inframe loss of 16 aa at protein level. As the exonic deletion can also be detected at the mRNA level, the finding implies a loss of TNAP function in this clone. The lost aa residues (human Tyr178 to Pro193) are evolutionary highly conserved and potentially interfere with TNAP activity. Clinically relevant examples for described variants reported within this position includes VAR_006156, at position 179; VAR_013982 at position 181; VAR_013983 at position 184; VAR_085157 at position 188; VAR_025917 at position 189; VAR_006157 at position 191; VAR_006158 at position 191 (Weiss et al., 1988; Taillandier et al., 2001; Di Mauro et al., 2002). AlphaFold Server predictions of this variant imply rearrangements in the AP domain of TNAP at proximity to the Mg^2+^ ion binding site at aa position 173 (Supplementary Figure S5C).

Clone 2.3 shows a 1 bp missense substitution and is predicted to result in an amino acid change at evolutionary conserved position. The corresponding tyrosine at position 28 is located at beginning of helix structure at surface of TNAP enzyme dimer, potentially essential to stabilize dimer. The detected tyrosine (Tyr; Y) to histidine (His; H) exchange results in biochemical changes, like polarity, hydrophobicity, a imidazole side chain, and size (Physiochemical distance, Grantham´s distance score (Grantham, 1974): 83 Sneath´s index (Sneath, 1966): 23; Epstein´s coefficient of distance (Epstein, 1967): 0,6). TNAP dimer prediction by Alphafold3 further hints to minor changes in an exterior helix motive (Supplementary Figure S5E), and structure prediction implies no significant change in corresponding helix structure but has only very low prediction quality at this position (pIDDT <50). AlphaMissense Pathogenicity score for this variant is 0.615 and indicates likely pathogenic changes. The corresponding region has been described to be affected in pathogenic variants of HPP patients, one example is an odonto-HPP patient showing a compound heterozygote variant c. [82T>G]; [1162T>C]; p. (Tyr28Asp) (Taillandier et al., 2001). Further reports describe additional affected HPP patients, which depict compound-heterozygote variants and variable clinical severity, for example, c. [83A>G]; [ = ]; p. (Tyr28Cys) Asymptomatic with biochemical phenotype, c. [83A>G]; [407G>A] Infantile pathogenic, c. [83A>G]; [746G>T] Infantile pathogenic, and c. [83A>G]; [98C>T] Infantile pathogenic (Taillandier et al., 2001; Liu et al., 2021).

Comparison to published databases (Farman et al., 2024) and structure predictions of *ALPL* genetic variants by computational modeling give indications if and how TNAP function might be affected. Bioinformatics tools and models for prediction of the severity of a respective mutation are reliable for well investigated cases, and for mutations located in regions encoding central functional domains, like the active center, as they lead to severe enzymatic defects (Silvent et al., 2014). Novel prediction tools like AlphaFold, AlphaMissense and AI guided pathogenicity prediction, nowadays envision fast classification of novel detected or less prominent patient variants by extrapolation to previous reported cases (Guzman-Vega et al., 2023; Jumper et al., 2021; Varadi et al., 2024). Although these predictions hold great potential to build biochemical hypothesis and to accelerate disease diagnostics, e.g., (Zhang et al., 2022; Cheng et al., 2023; Varadi et al., 2024), they often lack experimental consolidation for single variants. Considering the different ALPL variants induced in the newly established lines and the variable HPP phenotypes we decided to investigate cellular properties in more detail.

### Cellular properties of *ALPL* ^*tg*^ PDL-hTERT cells

Functional investigations of different *ALPL* ^*tg*^ PDL-hTERT cell lines showed heterogeneous results, by depicting differences in TNAP activity measurement via CSPD assays, cell proliferation assays, metabolic activity and Alizarin red staining of calcified tissues after *in vitro* osteogenic differentiation. Clone 1.1 and 1.3 lines inherit the same genetic variant but differ obviously in their cellular properties due to their heterozygote or homozygote *ALPL* variant status. The homozygous deletion detected in clone 1.1. resulted in a TNAP loss-of-function phenotype and showed maximal differences in measurements of several *in vitro* properties and after differentiation. Although both clones nearly show any residual TNAP expression, they both depict overall low TNAP activity rates and most times rather similar cellular properties. Other heterozygous clones with different genetic variants, like clone lines 1.2, 1.3, and 1.5 show similar CSPD assay values, similar TNAP residual activity and protein expression, but vary in their osteogenic differentiation potential.

Mitochondria are key energy source in cells, and their disruption leads to subsequent mitochondrial dysfunction and tissue destabilization and diseases. The essential function of ALPL/TNAP in mitochondria have recently been intensively studied and imply function in adaptive thermogenesis of fat cells and in regulation of ATP levels in bone, muscle progenitor, and cancer cells (Khatun et al., 2020; Sun et al., 2021; Zhang et al., 2021). Mitochondrial dynamics and mitophagy has been shown to be involved in cell mechanosensitivity and commitment of PDL cells (Zhang et al., 2023). However, detailed evidence on the role of mitochondria in PDL cells are still lacking. Assessment of TOMM20, mitochondrial markers has shown to be important for evaluation of reprogramming efficiency and ALPL expression by reprogrammed fibroblast (Prieto et al., 2016). In our study, unexpectedly, clone line 1.2. shows low expression of TOMM20 and ACTB in multiple Western blot analyses of whole cell lysates, which implies general disturbed mitochondrial dynamics. TOMM20 is detectable in MEFs samples from this line, which hints to a retained mitochondrial integrity but alterations in intracellular localization. Although we could not elucidate colocalization of TNAP and mitochondria, our results indicate that residual TNAP expression might origin from mitochondrial compartment of PDL-hTERT cells, and particularly in clone 2.3, which has shown the highest spontaneous TNAP activity within clones.

Mitochondrial oxidative phosphorylation (OXPHOS) is the main source of ATP. Thus, dividing skeletal progenitors and osteogenic commitment rely on mitochondrial OXPHOS (Lin et al., 2022). *Alpl* deficiency is shown to enhance ATP release and reduces ATP hydrolysis in mesenchymal stromal/stem cells (MSCs). Excessive extracellular ATP is, in turn, internalized by MSCs and causes increase in the intracellular ATP level, further regulating MSC differentiation (Liu et al., 2018). Our results in *ALPL* ^*tg*^ PDL-hTERT cells showed significantly low population doubling, low ATP levels, and significantly lower expression of complex II unit (succinate dehydrogenase (SDH)) in clone 1.5. Besides SDH, clone 1.5. displayed deficiency of all, ETC., complexes in their ME, which might explain reduced population doublings and ATP levels. Changes in succinate availability and SDH activity are shown to be involved in improved proliferation, migration, and osteogenesis of PDL cells (Mao et al., 2020). Thus, in comparison to these results, our study indicated that decreased SDH levels come from weakened cell mitochondrial fitness rather than succinate abundance. Further investigations are necessary to define precise relations between different genetic variants of *ALPL* and mitochondrial functionality.

Comparison of clone 2.3 to control PDL-hTERT cells strongly implies a rather normal phenotype, as this line depicts similar or even increased TNAP expression and functional levels. Cellular properties of clone 2.3 are not different to PDL-hTERT and wt cells in most experiments, e.g., CSPD activity, and even exceed these sometimes, e.g., during osteogenic differentiation and metabolic activity assays. *ALPL* expression and TNAP function have been reported to be regulated by the parathyroid hormone (PTH) (Wolf et al., 2013; Houston et al., 2016) and PTH stimulation during osteogenic differentiation of PDL-hTERT cells can also induce TNAP activity (Supplementary Figure S4A). PTH treatment of *ALPL* ^*tg*^ PDL-hTERT cells during long-term osteogenic differentiation up to 28 days show TNAP functional consequences on mineralization processes but did not alter their differentiation capacity (Supplementary Figures S4B,C).

**FIGURE 7:**
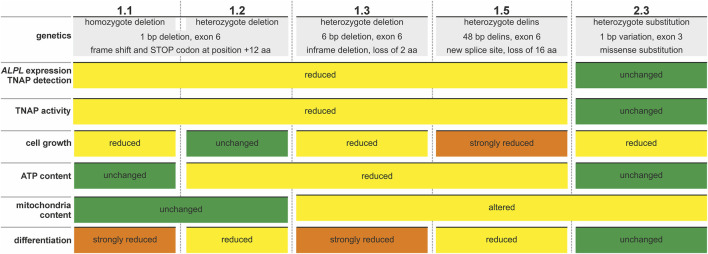
Summary of features detected in newly established *ALPL* ^*tg*^ PDL-hTERT cell lines. Colored plates indicate unchanged (green), changed or reduced (yellow) or strongly changed or reduced (orange) parameters in the different cell line clones.

Our current report describes the initial generation of the *ALPL* ^*tg*^ PDL-hTERT cell lines and establishes these as a novel *in vitro* model for investigation of dental HPP features (summary on cell line feature is given in Figure 7). The presented study focuses on investigation of TNAP function and lacks detailed functional assays for PDL-specific parameters. Future experiments with these cells therefore should include analyses of PDL-specific properties like cementum formation, as loss of primary tooth in HPP patients lack cement cementum (van den Bos et al., 2005; Zweifler et al., 2015). For this purpose a direct comparison of gene expression levels and cellular properties in the newly established *ALPL* ^*tg*^ PDL-hTERT cells lines to odonto-HPP patient-derived primary isolated, mesenchymal stem cells, or to ligament stem cells like PDLSC (Yang et al., 2024), or to pulp stem cells like hPDLSCs (Lee et al., 2022), is desirable. An additional point to clarify in future studies is how immortalization affects the PDL status of these cells. Constant *in vitro* propagation can alter cellular properties, like stemness, and may result in loss of differentiation potential (Zeng et al., 2023).

## Data Availability

The original contributions presented in the study are included in the article/Supplementary Material, further inquiries can be directed to the corresponding authors.
