# Supplementary material for: Establishment of human periodontal ligament cell lines with ALPL mutations to mimic dental aspects of hypophosphatasia
Source: Front Cell Dev Biol. 2025 Jun 3;13:1572571. doi: 10.3389/fcell.2025.1572571 (PMC12170583; doi:10.3389/fcell.2025.1572571)
Supplement: Supplementary file 1 [file DataSheet1.pdf]

Supplementary tables

Table S1: PCR Primers and DNA plasmids used in this study.

Primer name	Primer sequence (5´-3´)	Amplicon size [bp]	Purpose
Hu_ALPL1_gDNA_CleAs_fwd	GATAAAGCCAAACCCGCCC	388	Cleavage Assay construct 1 (133 bp and 255 bp)
Hu_ALPL1_gDNA_CleAs_rev	AGAGAAATCCCACAGTGCCT		
Hu_ALPL2_gDNA_CleAs_fwd	CTGGAGGATCTGGATGGCAC	541	Cleavage Assay construct 2 (200 bp and 341 bp)
Hu_ALPL2_gDNA_CleAs_rev	TTACAGAGCCATGCCCAGTG		
Hu_CDK4_gDNA_CleAs_fwd	GCACAGACGTCCATCAGCC	577	Cleavage Assay positive contr. (256 bp and 301 bp)
Hu_CDK4_gDNA_CleAs_rev	GCCGGCCCCAAGGAAGACTGG GAG		
hu_B2M_qPCR_fwd	GATGAGTATGCCGCCGTGT	105	qPCR (NM_004048.2), housekeeping, intron-spanning
hu_B2M_qPCR_rev	TGCGGCATCTTCAAACCTCC		
hu_ALPL/TNAP_qPCR_fwd	AGAACCCCAAAGGCTTCTTC	74	qPCR (BC021289.2), intron-spanning
hu_ALPL/TNAP_qPCR_rev	CTTGGCTTTTCCTTCATGGT		
TNSALP_543_for	GCCCTCTCCAAGACGTACAA	374	RT-PCR, *ALPL* exon 5 to exon 6
TNSALP_884_rv	CCATGATCACGTCAATGTCC		
Hu_CRELD2_f	TCGATCCAGAGTCCCTCCAC	424	Off-target gDNA sequencing
Hu_CRELD2_r	TAGTCCTCAGGGAGAAGCCG		
Hu_NCOR2_f	TGGCCAGACGGTCCCT	400	Off-target gDNA sequencing
Hu_NCOR2_r	CCCCTCATTTCACAGGACCC		
Hu_RNF168_f	TCGTCGGCAGCGTCCCAACAAA CACGCCATGGTT	483	Off-target gDNA sequencing
Hu_RNF168_r	GTCTCGTGGGCTCGGAGACGTG TTGGATGCTCCTG		
Hu_SVEP1_f	TCGTCGGCAGCGTCTCTGAAGTC TGGGGAAGGCT	757	Off-target gDNA sequencing
Hu_SVEP1_r	GTCTCGTGGGCTCGGCCACTGG CACCACATAGGAG		
Hu_TRIM42_f	TCGTCGGCAGCGTCCGTGCCCT TGGACTTTGTTG	439	Off-target gDNA sequencing
Hu_TRIM42_r	GTCTCGTGGGCTCGGCTTGGCA GCTCTTGGGTACA		
Hu_TSCC1_f	TCAGACACAGACCTCCTCATCA	393	Off-target gDNA sequencing
Hu_TSCC1_r	AACCCGTTTTCCACTAATCAGC		
Hu_WDR1_f	TCGTCGGCAGCGTCGAGACCCA CAACTCTTCCGG	420	Off-target gDNA sequencing
Hu_WDR1_r	GTCTCGTGGGCTCGGTCGCTGG CATAGAGTTAGCG		

Hu_WDR59_f	TCGTCGGCAGCGTCCACACTGC AGCTTTCCAACC	440	Off-target gDNA sequencing
Hu_WDR59_r	TCTCGTGGGCTCGGGGGAAGAA ACGAGGCTCACA		
Hu_ABCC8_f	GAGAGGGGTGGGGAAGAGTC	430	Off-target gDNA sequencing
Hu_ABCC8_r	GAAAGATGGGCCCCCACAG		
Hu_C3orf30_f	CAACCTAGACCAGTCAGGGACG	430	Off-target gDNA sequencing
Hu_C3orf30_r	CTGACCATCACTCTGTTCATGC		
Hu_C15orf41_f	TCGTCGGCAGCGTCGAAACCAC TGTCTTGGGCTG	391	Off-target gDNA sequencing
Hu_C15orf41_r	GTCTCGTGGGCTCGGGGCAGGA AAACAGGAAGCTT		
Hu_CHCHD2P8_f	CCGCGTGACCCCTCTA	386	Off-target gDNA sequencing
Hu_CHCHD2P8_r	TCAGCACCTCATTGAAACCC		
Hu_PTPRE_f	TCGTCGGCAGCGTCGAGGCCTT CTGTAGTTGGGT	484	Off-target gDNA sequencing
Hu_PTPRE_r	GTCTCGTGGGCTCGGCCCCTCC AATGACTGGTCTT		
Hu_Runx2_f	GAGTGGACGAGGCAAGAGTT	127	osteogenic differentiation, qPCR (NM_001024637.3)
Hu_Runx2_r	CTGTCTGTGCCTTCTGGGTT		
Hu_Sox9_f	GCAGGCCGACTCGCCACAC	73	osteogenic differentiation, qPCR (MN_000346.3)
Hu_Sox9_r	GGATTGCCCCGAGTGCTCGCC		
Hu_B3Galt2_f	GCCTGACCAAGGAAGAATGACTA	172	osteogenic differentiation, qPCR (NM_003783.3)
Hu_B3Galt2_r	TTGCAAAGCAGCAGTGTCTTC		
Hu_C5AR1_f	GAGACCAGAACATGGACTCCT	127	osteogenic differentiation, qPCR (NM_001736.4)
Hu_C5AR1_r	AGGATGTCTGGAACACGCAG		

Table S2: Amino acid sequences used for alignments and database identifiers.

UniProt ID	Protein name	Gene name, NCBI and ENSEMBL identifier	Species
P05186	PPBT_human;<br>Alkaline phosphatase, tissue-nonspecific isozyme	ALPL<br>NM_000478.6<br>ENSG00000162551	HS= human,<br>*Homo sapiens*
P10696	PPBN_human;<br>Alkaline phosphatase, germ cell type	ALPG (ALPPL, ALPPL2)<br>NM_031313.3<br>ENSG00000163286	HS= human,<br>*Homo sapiens*
P05187	PPB1_human;<br>Alkaline phosphatase, placental type	ALPP (PLAP)<br>NM_001632.5<br>ENSG00000163283	HS= human,<br>*Homo sapiens*
P09923	PPBI_human;<br>Intestinal-type alkaline phosphatase	ALPI<br>NM_001631.5<br>ENSG00000163295	HS= human,<br>*Homo sapiens*
K7B4Y6	PANTR Alkaline phosphatase (ALPL)	ALPL<br>ENSPTRG00000000302	PT= Chimpanzee,<br>*Pan troglodytes*
P09487	PPBT BOVIN Alkaline phosphatase tissue-nonspecific isozyme	ALPL<br>NM_176858.2<br>ENSBTAG00000008951	BT=Cow,<br>*Bos Taurus*
Q29486	PPBT FELCA Alkaline phosphatase tissue-nonspecific isozyme	ALPL<br>NM_001042563.1<br>ENSFCAT00000002960	FC=Cat,<br>*Felis catus*
P09242	PPBT MOUSE Alkaline phosphatase tissue-nonspecific isozyme	Alpl<br>NM_007431.4<br>ENSMUSG00000028766	MM= Mouse,<br>*Mus musculus*
P08289	PPBT RAT Alkaline phosphatase tissue-nonspecific isozyme	Alpl<br>NM_013059.3<br>ENSRNOG00000013954	RN= Rat,<br>*Rattus norvegicus*
Q92058	PPBT CHICK Alkaline phosphatase tissue-nonspecific isozyme	ALPL<br>NM_205360.2<br>ENSGALT00010051112	GG= Chicken,<br>*Gallus gallus*
F1Q5B5	DANRE Alkaline phosphatase (ALPL)	alpl<br>NM_201007.2<br>ENSDARG00000015546	DR= Zebrafish,<br>*Danio rerio*

Table S3: Off-target coding genes, ENSEMBL IDs, genomic position (hg38) and CFD off-target score (based on Doench, J., Fusi, N., Sullender, M. *et al.* *Nat Biotechnol* 34, 184–191 (2016))

ALPL CRISPR/Cas9 construct 1 – off-targets	*ALPL* CRISPR/Cas9 construct 2 – off-targets
CRELD2<br>ENSG00000184164, chr22:49919291-49919313<br>CFD off-target score: 0.014	*ABCC8*<br>ENSG00000006071, chr11:17397011-17397033<br>CFD off-target score: 0.000
NCOR2<br>ENSG00000196498, chr12:124486449-124486471<br>CFD off-target score: 0.055	*C3orf30 /RP11-484M3.5*<br>ENST00000490594, chr3:119146251-119146273<br>CFD off-target score: 0.071
RNF168<br>ENSG00000163961, chr3:196503123-196503145<br>CFD off-target score: 0.035	*C3orf30/HBEGF*<br>ENST00000230990, chr5:140342773-140342795<br>CFD off-target score: 0.056
SVEP1<br>ENSG00000165124, chr9:110365744-110365766<br>CFD off-target score: 0.133	*C15orf41*<br>ENSG00000186073, chr15:36708300-36708322<br>CFD off-target score: 0.007
TRIM42<br>ENSG00000155890, chr3:140688452-140688474<br>CFD off-target score: 0.018	*CHCHD2P8*<br>ENSG00000235115, chr13:28107647-28107669<br>CFD off-target score: 0.027
TSSC1<br>ENSG00000165699, chr2:3208397-3208419<br>CFD off-target score: 0.0125	*PTPRE*<br>ENSG00000132334, chr10:128066083-128066105<br>CFD off-target score: 0.106
WDR1<br>ENSG00000071127, chr4:10077385-10077407<br>CFD off-target score: 0.078	
WDR59<br>ENSG00000103091, chr16:74938191-74938213<br>CFD off-target score: 0.155	
EML4<br>ENSG00000143924, chr2:42325490-42325512<br>CFD off-target score: 0.139	

Supplementary data

Cleavage Assay

In vitro function of CRISPR/Cas9 components and transfection was controlled by T7-endonuclease cleavage assay according to manufacturer's protocols (GeneArt Genomic Cleavage Detection Kit (ThermoFisher Scientific, A24372). Fig. S1 depicts a representative assay result.

Cleavage efficiency was calculated by band densitometry of four independent experiments using ImageJ software (NIH, USA; https://imagej.net/software/fiji/) and indicated 12.73 % (SD = 5.00 %) efficiency for *ALPL1* crRNA, 15.97 % (SD = 6.95%) efficiency for *ALPL2* crRNA, and 35.88 % (single transfection) efficiency for CDK4 control crRNA.

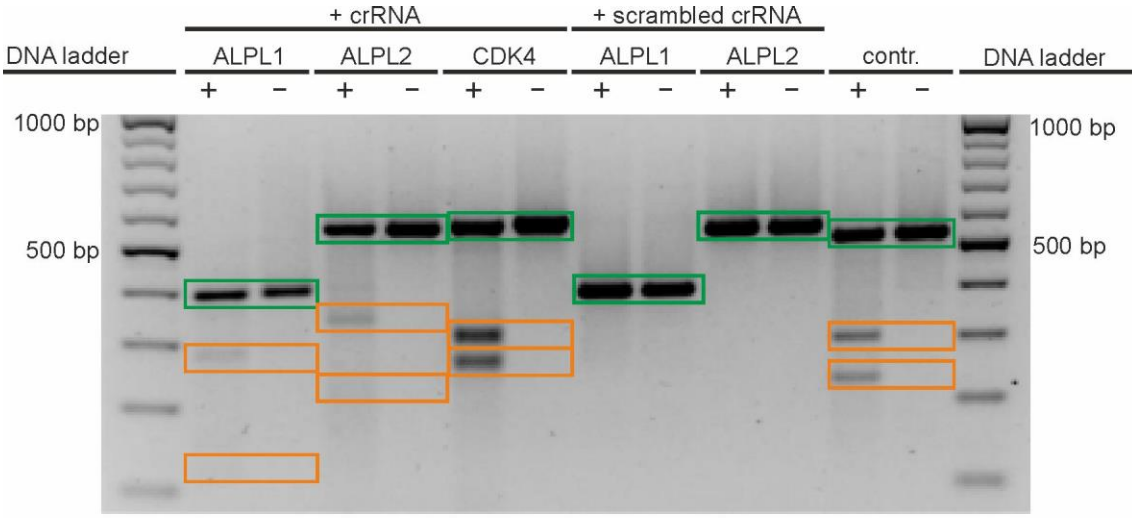

Fig. S1: Test of scRNA function and transfection by cleavage assays.

Evaluation of scRNA transfection and test for altered genomic DNA at the targeted regions was performed using a cleavage assay. The agarose gel depicts cleavage results of PDL-hTERT cells transfected either with *ALPL1*, *ALPL2*, *CDK4* (positive control), or scrambled crRNA. + samples were incubated, while – samples were not incubated with the T7 endonuclease enzyme. Green boxes mark the expected sizes of not altered product bands. Orange boxes mark the expected sizes of altered product bands, indicating the introduction of genomic alterations at the corresponding locus. Control lane depicts GeneArt® Genomic Cleavage Detection Kit reference PCR controls with and without T7 endonuclease enzyme incubation (product size: 516 bp, cleavage products: 291 and 225 bp).

CRISPR off-target prediction and testing

Sanger sequencing was performed to analyze potential exonic off-targets of used crRNA transfection constructs that were predicted by the tool CRISPOR (http://crispor.tefor.net). The results are summarized in Table S3.

ALPL construct 1 (Exon 4) shows 72 potential off-target sites, 9 in coding exons. The corresponding clone lines 1.1, 1.2, 1.3, and 1.5 were analyzed for mutations at these regions via Sanger sequencing (an example of sequencing results is given in Fig. S2). *ALPL* construct 2 (Exon 3) shows 73 potential off-target sites, 6 in coding exons. The corresponding clone line 2.3 was analyzed for mutations at these regions via Sanger sequencing (Fig. S3). No unwanted mutations were introduced by the gene editing procedure at the investigated off-target sites.

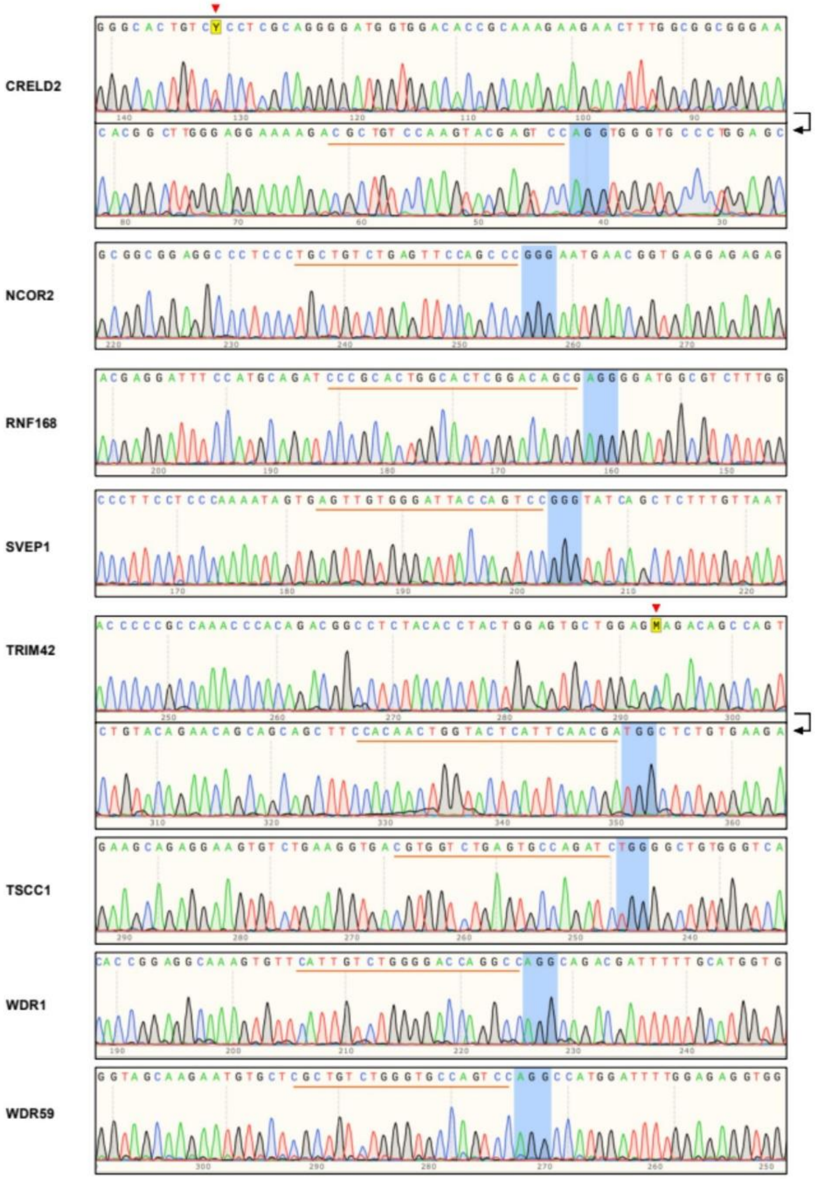

Fig. S2: Example of off-target sequencing results from *ALPL* construct 1.

Different genomic regions listed in table S3 were analyzed in clone line 1.3 via Sanger sequencing. Corresponding sgRNA target sites within these regions are underlined in orange and corresponding PAM sequences are marked in blue. Detected genetic variations are marked with red arrowheads and correspond to variants in the originally used PDL-hTERT cell line.

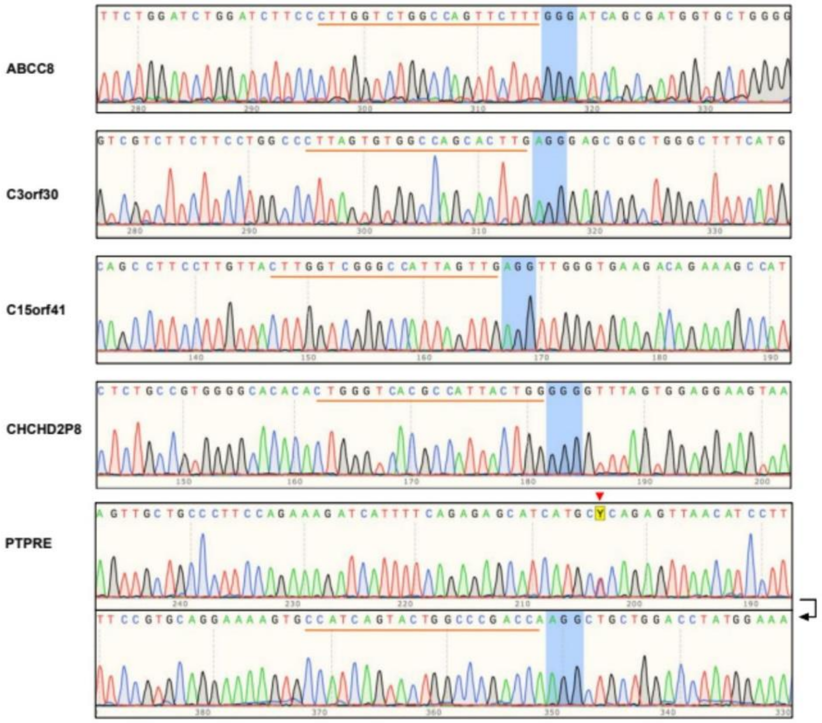

Fig. S3: Examples of off-target sequencing results from *ALPL* construct 2.

Different genomic regions listed in table S3 were analyzed in clone line 2.3 via Sanger sequencing. Corresponding sgRNA target sites within these regions are underlined in orange and corresponding PAM sequences are marked in blue. Detected genetic variations are marked with red arrowheads and correspond to variants in the originally used PDL-hTERT cell line.

PTH stimulation of clonal ALPL^{tg}-PDL-hTERT cells

For Parathyroid hormone (PTH) stimulation cells were grown under standard differentiation conditions in fibronectin coated well-plates to suppress cell detachment during differentiation (6-well plates for protein extraction, 24-well plates for Alizarin Red S staining). Cells were initially seeded at 20,000 cells/cm^3 density and incubated at 37°C.

For investigation of short-term PTH stimulation on TNAP activity in PDL-hTERT cells three wells per treatment were seeded and incubated for 4 days in differentiation medium (basal medium substituted with 10 mM β-Glycerophoshate (Sigma-Aldrich, G9422), 100 nM 2-Phospho-L-ascorbic acid trisodium salt (Sigma-Aldrich, 49752) and 100 nM Dexamethasone (Sigma-Aldrich, D4902)). For PTH stimulation the differentiation medium was exchanged with basal medium substituted with 10 nM or 50 nM PTH (human, fragment 1-34, Sigma-Aldrich, P3796) and incubation was performed until harvesting of cells after 1, 3 and 6 h. Controls were incubated in basal medium without PTH substitution. Specific TNAP activity was subsequently quantified by a CSPD assay (Fig. S4A)

For investigation of long-term PTH stimulation on PTH PDL-hTERT cell clones and corresponding controls were incubated for 6 h in basal medium substituted with 50 nM PTH after reaching confluency. Subsequently, PTH medium was exchanged with osteogenic differentiation medium, and incubation was prolonged for 42 h (Fig. S4B). Corresponding controls groups were incubated for 6 h in basal medium without PTH substitution. The 48 h PTH treatment cycle was repeated for an overall time frame of 22 and 28 days and was followed by Alizarin Red S staining to detect mineralization (Fig. S4C) and subsequently quantified (Fig. S4D).

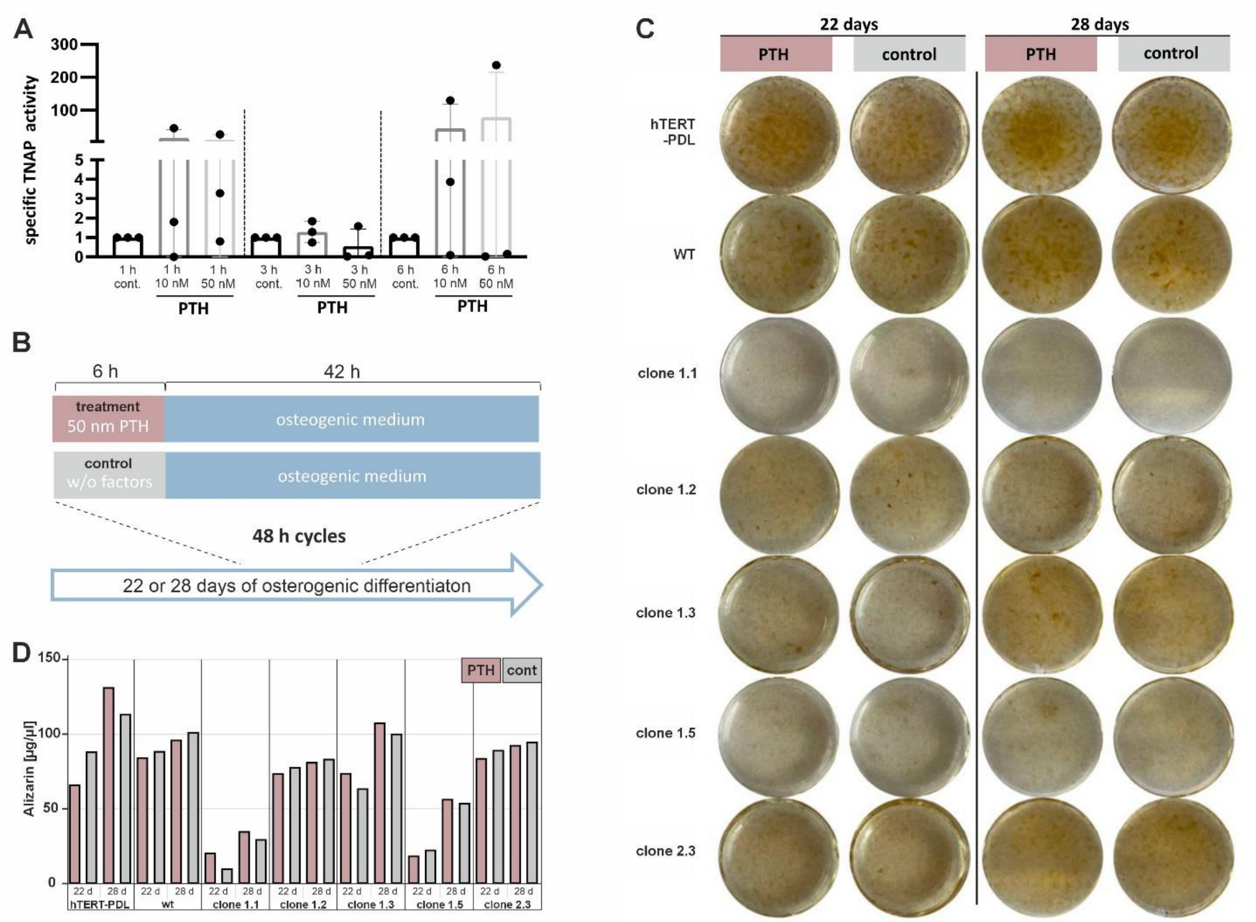

Fig. S4: Osteogenic differentiation of cell lines with or without intermittent PTH supplementation.

(A) Evaluation of the response of PDL-hTERT cells to stimulation with PTH (human, fragment 1-34, dilution in PBS) that has previously been shown to increase *ALPL* expression and TNAP activity. Both tested PTH concentrations showed a trend to increase TNAP enzyme activity upon 1h and 6 h stimulation as assessed by CSPD assays. Results are presented as mean ± SEM, N = 3 per time point and condition. (B, C) Mineralization capacity of *ALPL*^{tg} PDL-hTERT cell lines was tested after 22 or 28 days of incubation in osteogenic differentiation medium with or without intermittent PTH supplementation (B) by Alizarin Red S staining (C). A variable mineralization (brownish to red staining) potential mostly reflecting the TNAP expression level in the different cell lines was seen, while PTH stimulation did not cause an effect. (D) shows measurement of Alizarin levels in treated groups (mean values of three technical replicates for each treatment group are shown).

Alpha-Fold3 prediction models of *ALPL*^{tg}-hTERT-PDL clones

In silico prediction of altered TNAP 3D-structures caused by different genetic *ALPL* variants was performed by Alpha-Fold3 (https://alphafold.ebi.ac.uk/). Depicted structures in Fig. S5 show TNAP dimers visualized in SWISS-MODEL Workspace (https://swissmodel.expasy.org/) and are color coded for confidence levels of predicted structures. In addition, an overlay with TNAP dimer reference structures (colored in white/grey) was performed for investigation of altered protein folding.

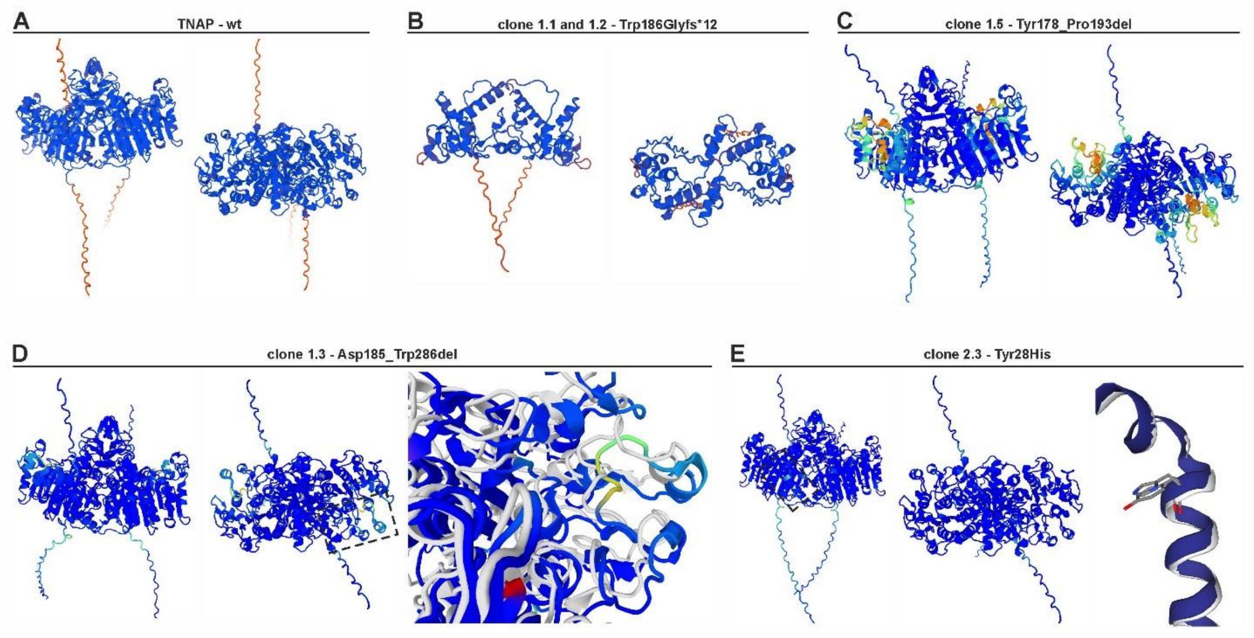

Fig. S5: AlphaFold 3 prediction of TNAP clone dimers.

AlphaFold Server prediction (Abramson et al., 2024) was used to model consequences of genetic alteration detected in different *ALPL*^{tg}-hTERT-PDL cell lines on TNAP dimer formation. Shown are (A) TNAP wildtype dimer (UniProt reference sequence P05186; PPBT_HUMAN), (B) clone 1.1 and 1.2 variant Trp186Glyfs*12, (C) clone 1.5 variant Tyr178_Pro193del, (D) clone 1.3 variant Asp185_Trp186del, (E) clone 2.3 variant Tyr28His. Visualization of AlphaFold predicted 3D structures was done by SWISS-MODEL Workspace. Displayed structure are rendered with PV viewer and show Cartoon representations of confidence levels (gradient and class). Higher magnification images in (D) and (E) focus on affected areas. Sequence of the corresponding clone is colored, while TNAP reference structures are depicted in white/grey. Dashed lines in D and E imply areas of higher magnification.

Investigation of relative gene expression in PDL-hTERT cells during osteogenic differentiation.

Differentiation timing of PDL-hTERT cells under osteogenic differentiation conditions was investigated by quantifying expression of several marker genes by qPCR (used primer pairs are listed in Table S1).

C5AR1 and B3Galt2 gene expression mark early phases of cell-fate determination (van de Peppel et al., 2017). The gene expression levels of Runx2 and Sox9 and its ratio were investigated as markers for early osteogenicity (Loebel et al., 2015). Col1a1 expression was investigated as a late osteogenic marker and marks connective tissue matrix production.

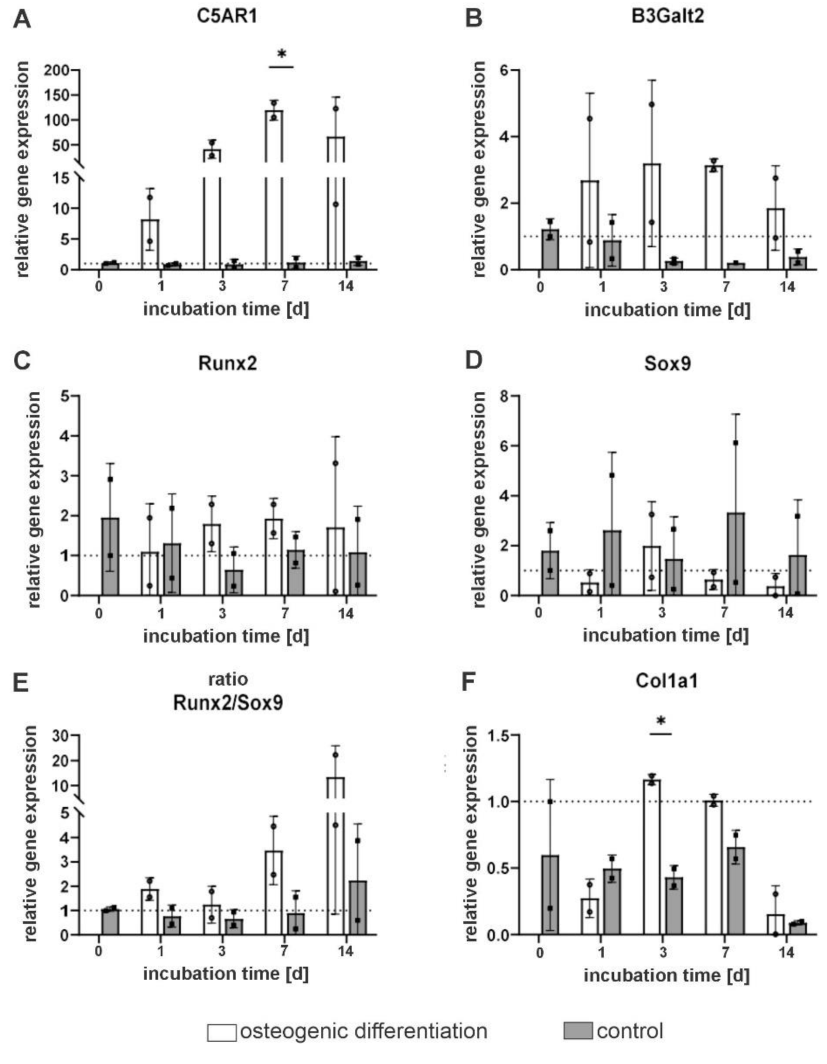

Fig. S6: Relative gene expression in PDL-hTERT cells during osteogenic differentiation.

Expression levels of C5AR1 (A), B3Galt2 (B), RUNX2 (C), SOX9 (D) and COL1A1 (F) were investigated via qPCR at different time points during in vitro osteogenic differentiation of PDL-hTERT cells (n=2). The ratio of RUNX2/SOX9 expression was calculated and is depicted in (E). Relative expression values were calculated by $2^{\Delta\Delta Ct}$ method and were normalized to day 0 samples. Statistical analyses were performed by ANOVA (including Dunnett correction) and unpaired t-test (including Holm-Sidak correction). Asterisks indicate $p < 0.05$. Whiskers indicate standard deviation.

Supplementary methods

Cell number determination and proliferation assay.

Cell number was determined prior to seeding via a Neubauer counting chamber. 10 µl cell suspension in PBS was mixed with 90 µl 0.4% Trypan blue (Gibco, 15250-061). Mean values of living cells were counted in four independent squares and correlated to the chamber dilution factor, e.g. for setting up comparable cell concentrations for in vitro experiments.

For measurement of cell proliferation $1*10^6$ cells were seeded and grown to confluency (approx. 5 days). Cell number was manually determined, and $1*10^6$ cells were reseeded. Population doubling time was calculated according to:

$$\frac{population\ doubling}{day} = \frac{\ln\left(\frac{cell\ number}{1*10^6}\right)*3.33}{days\ since\ last\ passage}$$

Alizarin staining and quantification

Cells were stained after 28 days of in vitro osteogenic differentiation with Alizarin red to visualize calcified ECM. Initially, culture medium was discarded, then cells were washed with PBS twice, fixed in 70% ethanol solution and stored at -20°C for 1h. Subsequently, cells were air-dried and incubated with staining solution (0.3 g Alizarin Red S ((Sigma Aldrich, A-3757) solution in 15 ml deionized water, pH 4.2). After 15-min incubation at RT on a shaker, samples were washed twice with deionized water. Imaging was performed with an upright microscope before quantification. For Alizarin red quantification, single wells were incubated with 10% cetylpyridinium chlorid solution (Sigma Aldrich, C07332, in Trisodium phosphate (pH 7.0)) for 20 min on a shaker. Corresponding Alizarin red suspensions were quantified on a Tecan Infinitive plate reader by measurement of absorbance at 570 nm (100 µl of each sample in triplicates in 96-well plates). Different Alizarin red dilutions in 10% cetylpyridinium chlorid solution were mixed to prepare reference standards. The dilution series ranged from 0 to 1000 µg/ml of Alizarin red dye and was subsequently used to calculate Alizarin red concentration in the corresponding cell samples.

Supplementary references

Abramson, J., Adler, J., Dunger, J., Evans, R., Green, T., Pritzel, A., Ronneberger, O., Willmore, L., Ballard, A.J., Bambrick, J., Bodenstein, S.W., Evans, D.A., Hung, C.C., O'neill, M., Reiman, D., Tunyasuvunakool, K., Wu, Z., Zemgulyte, A., Arvaniti, E., Beattie, C., Bertolli, O., Bridgland, A., Cherepanov, A., Congreve, M., Cowen-Rivers, A.I., Cowie, A., Figurnov, M., Fuchs, F.B., Gladman, H., Jain, R., Khan, Y.A., Low, C.M.R., Perlin, K., Potapenko, A., Savy, P., Singh, S., Stecula, A., Thillaisundaram, A., Tong, C., Yakneen, S., Zhong, E.D., Zielinski, M., Zidek, A., Bapst, V., Kohli, P., Jaderberg, M., Hassabis, D., and Jumper, J.M. (2024). Accurate structure prediction of biomolecular interactions with AlphaFold 3. *Nature* 630**,** 493-500.

Loebel, C., Czekanska, E.M., Bruderer, M., Salzmann, G., Alini, M., and Stoddart, M.J. (2015). In vitro osteogenic potential of human mesenchymal stem cells is predicted by Runx2/Sox9 ratio. *Tissue Eng Part A* 21**,** 115-123.

Van De Peppel, J., Strini, T., Tilburg, J., Westerhoff, H., Van Wijnen, A.J., and Van Leeuwen, J.P. (2017). Identification of Three Early Phases of Cell-Fate Determination during Osteogenic and Adipogenic Differentiation by Transcription Factor Dynamics. *Stem Cell Reports* 8**,** 947-960.
